# RIP-seq analysis of eukaryotic Sm proteins identifies three major categories of Sm-containing ribonucleoproteins

**DOI:** 10.1186/gb-2014-15-1-r7

**Published:** 2014-01-07

**Authors:** Zhipeng Lu, Xiaojun Guan, Casey A Schmidt, A Gregory Matera

**Affiliations:** 1 Departments of Biology and Genetics, Integrative Program for Biological and Genome Sciences, University of North Carolina, Chapel Hill, NC 27599-3280, USA; 2 Curriculum in Genetics & Molecular Biology, University of North Carolina, Chapel Hill, NC 27599-3280, USA; 3 Center for Bioinformatics, University of North Carolina, Chapel Hill, NC 27599-3280, USA; 4 Current address: Sequenom, San Diego, CA 92121, USA

## Abstract

**Background:**

Sm proteins are multimeric RNA-binding factors, found in all three domains of life. Eukaryotic Sm proteins, together with their associated RNAs, form small ribonucleoprotein (RNP) complexes important in multiple aspects of gene regulation. Comprehensive knowledge of the RNA components of Sm RNPs is critical for understanding their functions.

**Results:**

We developed a multi-targeting RNA-immunoprecipitation sequencing (RIP-seq) strategy to reliably identify Sm-associated RNAs from *Drosophila* ovaries and cultured human cells. Using this method, we discovered three major categories of Sm-associated transcripts: small nuclear (sn)RNAs, small Cajal body (sca)RNAs and mRNAs. Additional RIP-PCR analysis showed both ubiquitous and tissue-specific interactions. We provide evidence that the mRNA-Sm interactions are mediated by snRNPs, and that one of the mechanisms of interaction is via base pairing. Moreover, the Sm-associated mRNAs are mature, indicating a splicing-independent function for Sm RNPs.

**Conclusions:**

This study represents the first comprehensive analysis of eukaryotic Sm-containing RNPs, and provides a basis for additional functional analyses of Sm proteins and their associated snRNPs outside of the context of pre-mRNA splicing. Our findings expand the repertoire of eukaryotic Sm-containing RNPs and suggest new functions for snRNPs in mRNA metabolism.

## Background

Sm proteins are a family of highly conserved RNA-binding proteins present in all three domains of life [1,2]. In bacteria and archea, Sm homologs form either homohexameric (for example, Sm2 and Hfq) or homoheptameric (Sm1) ring-shaped complexes [3,4]. These complexes regulate the stability and translation of mRNAs by facilitating base pairing interactions between small RNAs (sRNAs) and mRNAs [5-7]. In eukaryotes, more than 20 Sm protein homologs assemble into several distinct heteroheptameric rings [8]. There are two major eukaryotic Sm classes: the canonical Sm proteins and the Sm-like (Lsm) proteins [9]. Canonical Sm proteins also form heptamers that bind the major and minor uridine-rich small nuclear ribonucleoprotein (snRNP) particles (U1, U2, U4, U4atac, U5, U7, U11 and U12). These small RNPs carry out important metabolic reactions such as pre-mRNA splicing and 3′ end processing [9-13]. Lsm proteins form two distinct heteroheptameric complexes. The Lsm1-7 ring directly binds the 3′ end of oligoadenylated mRNAs and is involved in regulating mRNA decay [14], while the Lsm2-8 ring binds to the 3′ oligouridine tail of U6 and U6atac small nuclear (sn)RNAs to form RNP particles that participate in pre-mRNA splicing [15-18]. Thus, the Lsm proteins, which regulate mRNA stability, are thought to be more akin to their archaeal and bacterial brethren.

A growing body of evidence points to potential new roles for canonical Sm proteins and Sm class snRNPs outside of the spliceosome in the processing, localization and translational control of messenger RNPs (mRNPs). In *Caenorhabditis elegans*, Sm proteins, but not other splicing factors, localize to germline P granules and are required for their integrity [19,20]. In *Drosophila melanogaster*, SmB and SmD3 are enriched at the posterior pole of developing oocytes [21,22], and a hypomorphic mutation in SmD3 causes mislocalization of *oskar* mRNPs and pronounced defects in germ cell specification that are independent from splicing [21]. Moreover, loss of the Sm protein methyltransferase PRMT5 results in failure to specify the germline [21,23,24]. Furthermore, a genetic screen for modifiers of FMR1 (Fragile X mental retardation 1) in *Drosophila* identified SmD3 as a suppressor of dFMR1’s translational repression function, and SmD3 and dFMR1 were found to colocalize within neuronal mRNP granules [25]. In vertebrates, Sm proteins are enriched in the nuage and mitochondrial cement [26,27], structures that share many components with the invertebrate germ plasm. The U1 snRNP, in addition to its splicing role, protects pre-mRNA from premature polyadenylation at cryptic poly(A) signals in introns [11,12,28], and inhibits HIV RNA polyadenylation [29,30]. In addition, RNA sequence elements complementary to the U1 5′ end play important roles in the stabilization of promoter-downstream transcripts and thus contribute to promoter directionality [31,32]. The U1 snRNP not only regulates gene expression via RNA processing; a modified form of U1 can also target HIV RNA to reduce viral protein expression [33]. Moreover, the U2 and U12 snRNPs play an unexpected role in promoting U7-snRNP-dependent processing of intronless histone mRNAs in human cells, and both protein-RNA interaction and RNA-RNA base-pairing suffice for the activity [34]. Collectively, these studies suggest additional functions for Sm proteins and snRNPs in RNA metabolism; however, little is known about the *in vivo* RNA targets that might be regulated by Sm proteins/snRNPs, in these processes.

To systematically identify Sm protein-containing RNPs, we carried out RNA-immunoprecipitation (RIP) against multiple Sm proteins from *Drosophila* ovaries and HeLa cells, followed by high-throughput sequencing (RIP-seq) of the immunopurified RNAs. Using this robust and reproducible multi-targeting RIP-seq approach, we recovered most of the spliceosomal snRNAs. In addition, we discovered a new *Drosophila*-specific snRNA, many Sm-associated small Cajal body-specific RNAs (scaRNAs), and numerous Sm-associated mRNAs from both *Drosophila* and human cells. The new snRNA is highly conserved in the melanogaster group of Drosophilids, although it is not essential for organismal viability. Two major categories of the Sm-associated mRNAs encode mitochondrial and translation-related proteins. Using quantitative reverse transcriptase PCR (qRT-PCR), we found that some of the RNA-Sm interactions are tissue-specific, whereas others are more widespread. The Sm-associated mRNAs are properly spliced and polyadenylated, indicating that the mRNA-Sm interactions reported here are distinct from those involved in pre-mRNA splicing and Lsm1-7 dependent degradation. We also provide evidence that the mRNA-Sm association is mediated by snRNPs, and we show that a predicted U1 snRNP base pairing region on an mRNA is required for interaction with this snRNP. These mature mRNA-snRNP interactions are very stable and distinct from other previously studied interactions (pre-mRNA splicing, ‘telescripting’ and regulation of promoter directionality). Taken together, the data identify additional direct targets of canonical Sm proteins, and suggest that Sm class snRNPs may have novel, evolutionarily conserved functions in mRNA localization, stability and translation.

## Results

### Identification of RNAs that co-purify with eukaryotic Sm proteins

As mentioned above, the Sm and Sm-like proteins comprise a family of ancient evolutionary origin that functions to modulate the stability and translation of several classes of RNA, including mRNAs [1,35]. Based on these ancestral roles, the involvement of eukaryotic Sm proteins in splicing is generally thought to be a derived function, and additional RNA targets of Sm proteins remain to be discovered.

**Figure 1:**
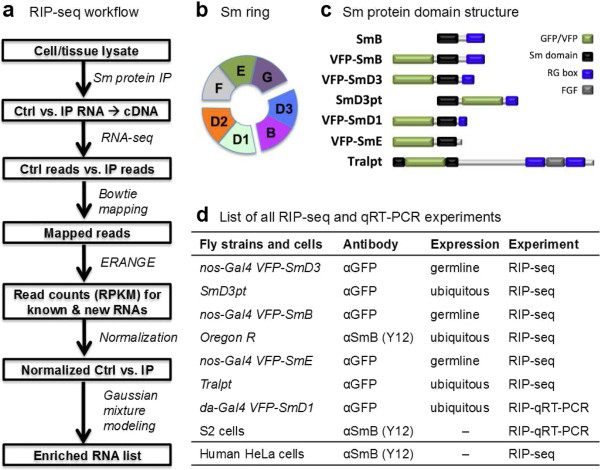
**RIP-seq experimental analysis strategies. (a)** Outline of RIP-seq analysis pipeline. See Materials and methods for details. **(b)** Schematic diagram of the canonical Sm ring. The three sub-complexes are shown separately. **(c)** Schematic diagram of the Sm-domain-containing proteins used in this study. **(d)** Summary of the RIP-seq and RIP-qRT-PCR experiments performed, targeting all three sub-complexes of the canonical Sm ring and Tral. See Table S1 in Additional file 1 for details. Ctrl, control; GFP, green fluorescent protein; IP, immunoprecipitation; RPKM (reads per kilobase per million reads); VFP, Venus fluorescent protein.

To characterize the repertoire of RNA targets that are associated with Sm proteins in *Drosophila* ovarian lysates, we performed RIP-seq analysis of individual subunits of the canonical Sm ring. We also performed RIP-seq on Trailer Hitch (Tral), a protein that contains an Sm domain (Figure 1c). Tral is not incorporated into the canonical Sm ring; therefore, we expected it to associate with a distinct subset of transcripts [36]. An outline of the experimental strategy and data analysis pipeline is shown in Figure 1a. Immunoprecipitations (IPs) were carried out using either anti-SmB (monoclonal antibody Y12) or anti-green fluorescent protein (anti-GFP) antibodies (for the GFP- and Venus fluorescent protein (VFP)-tagged proteins). Normal goat serum was used as control for the IP. Immunoprecipitated RNA was reverse transcribed to cDNA, fragmented, ligated with adapters, PCR-amplified and sequenced on an Illumina Genome Analyzer II.

To reduce potential non-specific interactions and artifacts, we carried out RIP-seq on several Sm proteins expressed from three different genomic contexts: (i) native endogenous genes, (ii) VFP-tagged transgenes, or (iii) a gene-trapped (GFP-tagged) endogenous gene (Figure 1c). Comparisons among this wide variety of experimental conditions helps to minimize problems associated with genetic background, transgene overexpression, and antibody specificity. Four different transgenic lines were employed, including VFP-tagged SmD3, SmB, SmD1 and SmE [21]. Transgenes were expressed using the UAS/Gal4 system, crossed to a *nanos*-Gal4 driver for germline-specific expression or, in the case of VFP-SmD1, to a *daughterless*-Gal4 driver for ubiquitous expression [37]. SmB and SmD3 form an obligate dimer (Figure 1b), whereas SmD1 and SmE are present in distinct sub-complexes within the heteroheptameric ring structure [9]. Thus, IPs targeting different components of the Sm ring further reduced potential artifacts resulting from epitope tagging, as these proteins form a complex that is expected to bind a similar set of RNAs. RIP-seq experiments were performed on SmB, SmD3 and SmE, whereas RIP-qRT-PCR was performed on VFP-SmD1 for identified targets. To broaden the scope of our study, we also performed RIP-seq analysis in cultured human HeLa cells, using the Y12 antibody mentioned above (Figure 1d; see details in Table S1 in Additional file 1).

### Enrichment analysis of Sm RIP-seq experiments

We obtained between 8 and 28 million 35-nucleotide single-end reads per *Drosophila* ovary RIP-seq library, and roughly 20 million 48-nucleotide paired-end reads per human HeLa cell RIP-seq library. All of the fly and human sequencing data are of high quality (Figure S1 in Additional file 1). Despite differences in total read numbers, the IPs consistently yielded many more mappable reads than did the controls (Table S2 in Additional file 1, ‘mapped’ and ‘%mappable’ columns). This was to be expected; due to the low amount of input cDNA, most of the reads in the control IPs are not mappable (for example, rRNAs, primer/adapter dimers or even random sequences; Table S3 in Additional file 1) and those that do map to the genome typically correspond to abundant RNAs that stick to the beads non-specifically Library statistics show that random hexamer priming yielded more mappable reads than did oligo(dT)_20_ priming (Table S4 in Additional file 1). Thus, we used the random hexamer-primed libraries for the subsequent enrichment analyses.

We built a data analysis pipeline (Figure 1a) by integrating previously published programs (see Materials and methods for details). Sequence reads for the *Drosophila* RIP-seq experiments were mapped to the *Drosophila* expanded genome and quantified using ERANGE [38]. Then, for each experiment, we filtered out transcripts with read coverage less than 10. Assuming that the majority of RNA species are not associated with Sm proteins, we normalized the remaining transcripts against the median of all enrichment ratios: (raw_IP + 2)/(raw_Ctrl + 2). After normalization, we defined the enrichment ratio as (norm_IP + 2)/(norm_Ctrl + 2). The use of median-normalized raw read numbers is similar to the upper-quartile normalization method used by others [39]. In this way, we made a conservative estimate of the enrichment of RNAs in IPs versus controls.

**Figure 2:**
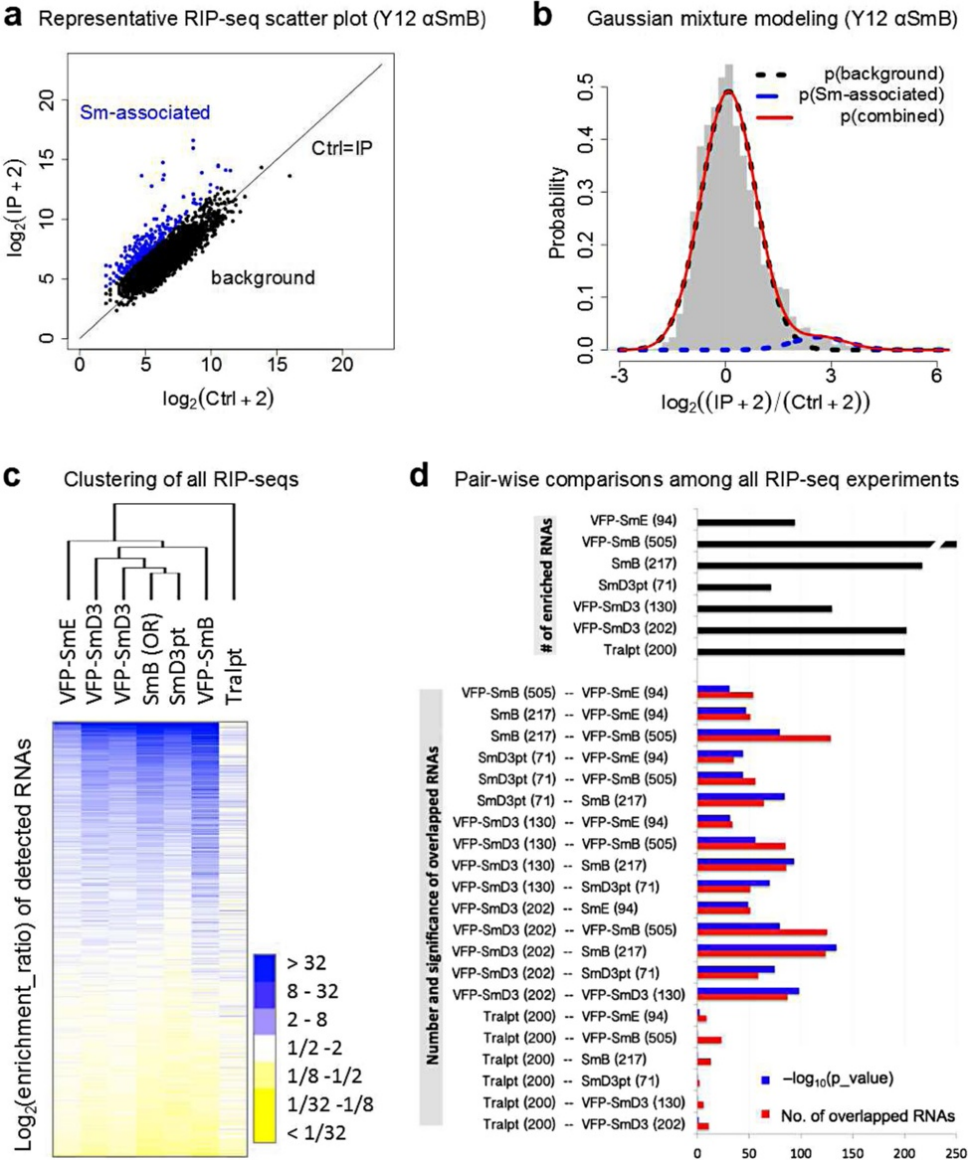
**RIP-seq data analysis. (a)** Scatterplot of a control (Ctrl)-IP pair of RIP-seq data (SmB IP Lu023-Lu024), where normalized and log-transformed read numbers for each known transcript in an IP are plotted against that of Ctrl (Ctrl + 2 and IP + 2 to avoid division by zero). Black dots represent background RNAs, while the blue dots represent enriched RNAs, as determined by Gaussian mixture modeling. Only RNAs with read coverage >10 are plotted. See Figure S1 in Additional file 1 for the rest of the scatterplots. **(b)** Gaussian mixture modeling of the RIP-seq data (SmB IP), where the enrichment ratios for all the transcripts were plotted as a histogram (in gray) and fitted with a combination of two Gaussian curves. **(c)** Log-transformed enrichment ratios of the 5,296 RNAs (with coverage d >10) in all 7 experiments were clustered (average linkage clustering using correlation (uncentered) as similarity metric) and visualized as a heat map. **(d)** Pair-wise comparisons among all seven experiments. Numbers of enriched RNAs are listed next to the experiment labels. Black bars, number of enriched RNAs in each experiment; red bars, number of overlapped RNAs in each pair; blue bars, negative log_10_ transformed Fisher’s exact test *P*-values (within a superset of 5,296 RNAs). See Figure S2 in Additional file 1 for pairwise comparisons excluding non-coding RNAs.

To visualize the enrichment data, scatter plots were constructed using the log-transformed and normalized read numbers. Data for the native SmB-associated RNAs (Oregon R, Y12 IPs) are shown in Figure 2a; data for the other Sm protein constructs are presented in Figure S1 in Additional file 1. In any co-IP experiment, there are two populations of molecules: those that interact specifically with the antibody and those that stick non-specifically to the beads. Non-specific interaction was observed for many transcripts, as depicted by the main cluster along the diagonal line (Figure 2a). The dots located above the main cluster represent the enriched RNAs. In order to objectively identify Sm-associated RNAs, we employed Gaussian mixture modeling [40], which has been used to analyze RIP-chip experiments [41]. Distributions of the enrichment ratios were first plotted as histograms. Next, we used mixtools to fit a combination of two Gaussian functions to the enrichment ratio distribution [42].

As shown in Figure 2b, the distribution of the log-transformed enrichment ratios (red line) can best be explained by two different Gaussian functions, one that corresponds to the background RNAs (black dotted line) and one that represents the Sm-associated RNAs (blue dotted line). The cutoff between Sm-associated and background mRNAs was defined by the log of the odds (LOD) ratio between the two Gaussian functions. The transcripts with a LOD > 1 (that is, those that had a greater likelihood of being in the Sm distribution) were considered to be Sm-associated RNAs. Using this threshold, we then mapped these assignments back onto the scatter plots. As shown in Figure 2a (blue dots), the enriched RNAs are clearly seen to be above the diagonal (black dots represent the background distribution). This same analysis was performed on the other Sm protein datasets, with strikingly similar results (Figure S2 in Additional file 1). Thus, the Gaussian mixture modeling procedure provides an unbiased and less arbitrary method for identifying enriched RNAs [41]. Using the aforementioned analysis pipeline, we identified roughly 200 Sm-associated RNAs in any given RIP-seq experiment, representing 0.7% of the *Drosophila* transcriptome, or 4% of the significantly expressed transcripts.

### A multi-targeting RIP strategy identifies highly reproducible Sm-associated RNAs

To assess the robustness and reproducibility of the *Drosophila* RIP-seq experiments and analysis pipeline, we visualized the log-transformed enrichment ratios for the transcripts with a read coverage greater than 10. Out of the >15,000 annotated genes in the fruitfly genome, 5,296 of them showed sufficient read depth (d > 10). To determine the relationship between the profiles of the seven RIP-seq experiments without prior assumptions, we performed an unsupervised hierarchichal clustering analysis. The top of the map represents RNAs that are significantly enriched (Figure 2c). As shown by the dendrogram (Figure 2c) and consistent with expectation, the six canonical Sm protein RIP-seq experiments clustered together, whereas the data from the Tral IP formed an outgroup. The most-highly enriched transcripts among the random hexamer-primed libraries from six Sm IP experiments (including one VFP-SmD3 biological replicate) revealed extensive overlap. Detailed analysis showed that 25 RNAs (9 snRNAs, 16 mRNAs) were common among all 6 Sm protein IPs, and 52 transcripts (12 snRNAs, 40 mRNAs) were shared in 5 of the 6 (see Table S5 in Additional file 1 for detailed enrichment ratios). The top 86 transcripts (13 snRNAs, 1 small nucleolar RNA (snoRNA), and 72 mRNAs) were shared by at least 4 of the experiments. Since four *Drosophila* snRNAs (U1, U2, U4, and U5) have multiple variant paralogs, we reassigned uniquely mappable reads to them and we found that all of the snRNAs with significant coverage are enriched in all Sm IPs (Table S6 in Additional file 1). In addition, we analyzed the consensus set of 86 Sm-associated RNAs in the oligo(dT)_20_ primed libraries, and we found that they are also highly enriched, despite the lower number of mappable reads (Figure S4 in Additional file 1). Thus, our multi-targeting RIP-seq approach is robust despite the differences in library statistics (Table S2 in Additional file 1). We operationally defined the Sm-associated RNAs as being those that were enriched in at least four of the six experiments.

Next, we carried out pair-wise comparisons among the seven RIP-seq experiments and performed Fisher’s exact test to assess the significance of any overlapping subsets (Figure 2d). Interestingly, among the top 200 RNAs in the Tral IP experiment, very few of them overlapped with any of the RNAs that associated with canonical Sm proteins. As seen in the heat map (Figure 2c), the enrichment ratios for the VFP-SmE IP were typically lower than those of the other Sm proteins. However, the pairwise comparisons show that SmE associates with a similar group of RNAs (see also Figure S4 in Additional file 1). The overlaps between the different Sm protein IPs were highly significant, as shown by their extremely small *P*-values (10^-32^ to 10^-135^, plotted as negative logarithms; Figure 2d). Even when all of the snRNAs were taken out of the pair-wise comparisons, the *P*-values remained extremely small (Figure 2d; Figure S3 in Additional file 1). Despite the different experimental parameters (tagged versus untagged, native versus ectopic, and so on), the lists of enriched RNAs are essentially the same. This high degree of reproducibility suggests that the multi-subunit targeting approach is superior to the conventional biological replication of experiments for RNP analysis. Indeed, the variability between biological replicates was greater in the case of VFP-SmD3 than it was between some of the other RIPs (Figure 2c). Collectively, these data demonstrate a high degree of specificity in the Sm protein IPs, showing that canonical Sm proteins co-precipitate with essentially the same set of mRNAs.

### Sm proteins associate with three major classes of RNAs

**Figure 3:**
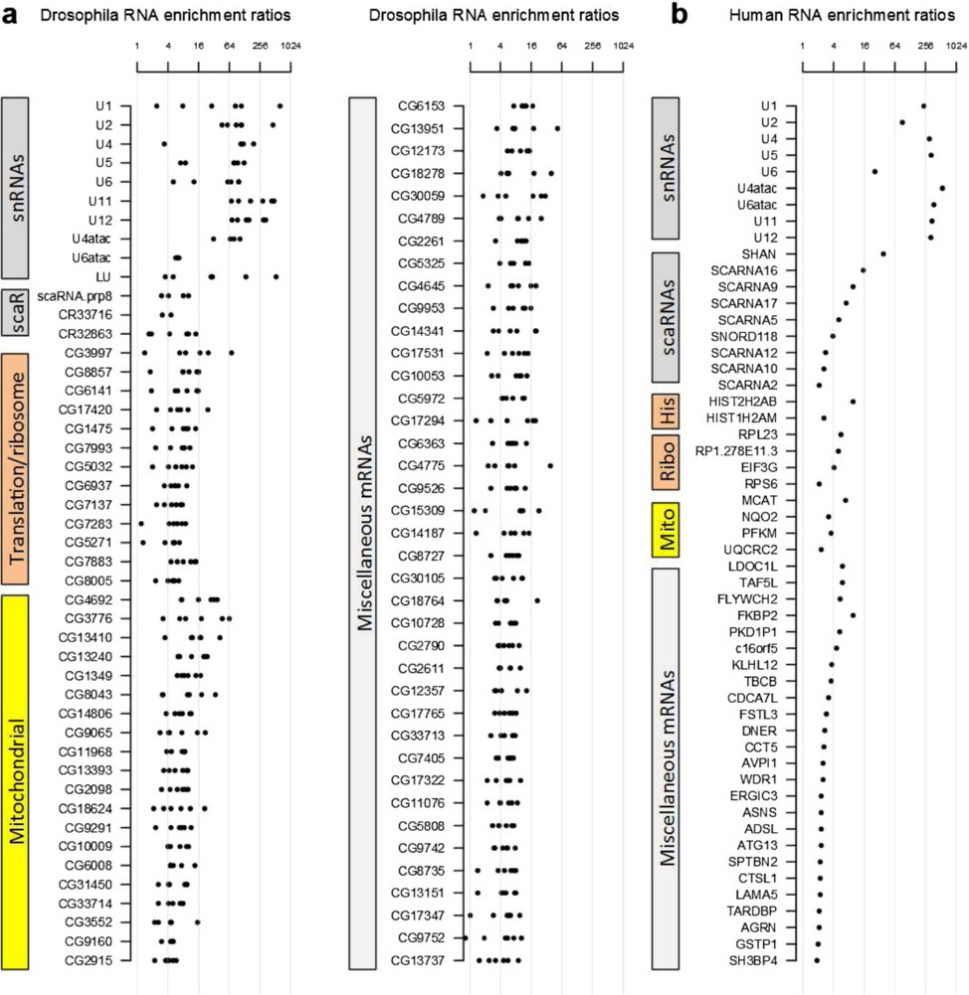
**Three categories of Sm-associated RNAs in *Drosophila *and human.** Different categories of Sm-associated RNAs are color-coded. **(a) ***Drosophila* Sm-associated RNAs, with enrichment ratios from all six Sm RIP-seq experiments. For snRNAs with multiple distinct paralogs (U1, U2, U4 and U5), all the reads were pooled for calculation of enrichment ratios. The three U6 paralogs are identical in sequence. See Table S6 in Additional file 1 for assignment of reads to distinct paralogs. U7 was not plotted due to low read coverage. See Table S5 in Additional file 1 for detailed enrichment ratios. **(b)** Human Sm-associated RNAs. Medians of enrichment ratios were plotted for snRNAs with multiple paralogs. See Table S7 in Additional file 1 for detailed enrichment ratios.

**Figure 4:**
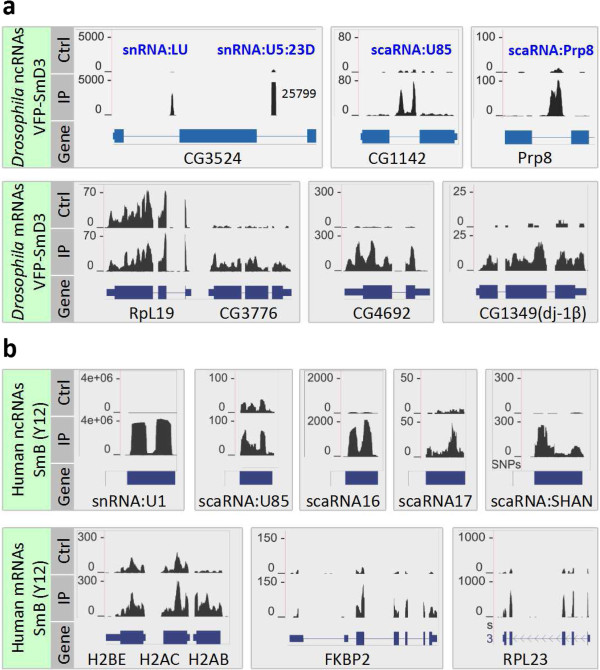
**Examples of the three categories of Sm-associated RNAs in *Drosophila *and human.** For genes with multiple transcripts, the gene model that is most similar to the read coverage pattern is shown. The y-axis corresponds to the normalized number of reads per nucleotide. **(a)** Examples of *Drosophila* Sm-associated RNAs from VFP-SmD3, control (Ctrl; Lu003) and IP (Lu004). For the non-coding RNAs that are associated with Sm proteins, their host genes are also shown. The read coverage for U5:23D is off scale, and thus truncated. **(b)** Examples of human Sm-associated RNAs from Y12 αSmB, Ctrl (Lu045) and IP (Lu047). The histone mRNAs H2BE, H2AC and H2AB are short for HIST2H2BE, HIST2H2AC and HISTH2AB, respectively.

The RIP-seq experiments in both *Drosophila* and human cells confirmed the well-studied snRNAs as major targets of Sm proteins, and in addition indicate novel classes of Sm targets. A detailed analysis of the known and newly discovered RNAs from our study suggests that Sm proteins associate with three major classes of RNAs (Figures 3 and 4; Figures S4 and S6 in Additional file 1).

#### RIP-seq identifies Sm class snRNAs

The Sm-associated transcripts and their enrichment ratios are listed in Figure 3. As expected, all spliceosomal snRNAs were among the top-scoring transcripts in terms of their enrichment ratios. The only missing Sm class snRNA from the list of Sm-associated RNAs is U7 snRNA, because it is too short (71 nucleotides in *Drosophila*, and 63 nucleotides in human) to be included in the size-selected cDNA libraries (Figure 3a; Table S5 in Additional file 1) [43,44]. Other highly abundant non-coding RNAs (ncRNAs; for example, 7SK snRNA, SRP RNA, 5.8S ribosomal RNA and so on, data not shown) were not enriched in the IPs, demonstrating the specificity of the approach. Multiple distinct paralogs exist for four of the *Drosophila* snRNAs, U1, U2, U4 and U5, and they share long stretches of identical regions (Figure S5 in Additional file 1). In order to accurately analyze each paralog without the confounding repetitive reads, we reassigned uniquely mappable reads to U1, U4 and U5 paralogs (Table S6 in Additional file 1). We used the variant nucleotides in U2 to calculate the fractions of each isoform and redistribute the total number of U2 reads among the gene paralogs. Not surprisingly, all snRNAs with significant read coverage are enriched in the IPs (Table S6 in Additional file 1). With regard to the HeLa cell analysis, there are hundreds of snRNA genes in the human genome, and only a small fraction of them are properly annotated. Not surprisingly, most of the annotated human spliceosomal snRNAs were identified in our IPs, all of which have very high enrichment ratios (Figure 3b).

ERANGE analysis and manual inspection of the *Drosophila* RIP-seq data revealed several clusters of reads that could not be mapped to gene models. Four of them are new genes that had not been previously annotated. During preparation of this manuscript, two transcriptomic studies have since identified these putative new transcripts [45,46]: CR43708, CR43600, snoRNA:2R:9445410 (CR43574) and snoRNA:2R:9445205 (CR43587). Two of the four novel transcripts, CR43708 and CR43600, showed significant enrichment in the IPs.

**Figure 5:**
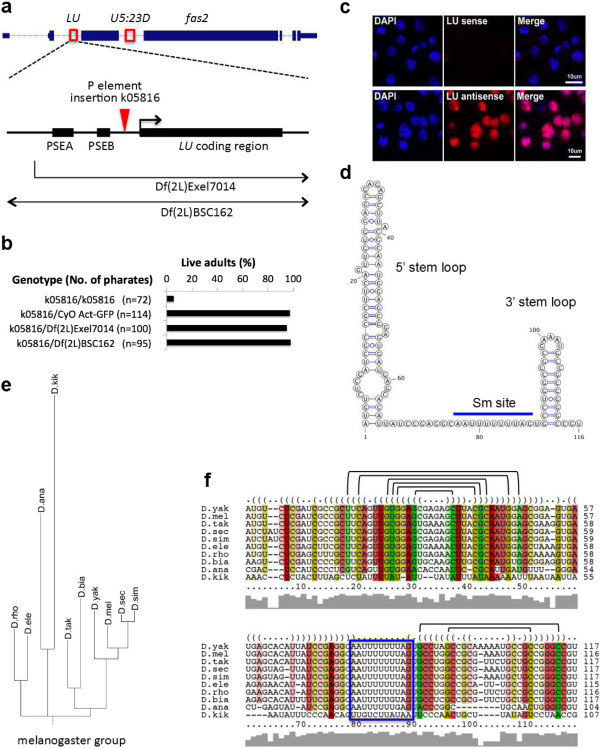
**Characterization of the *Like-U (LU) *snRNA gene. (a)** Genomic and genetic contexts of the *LU* snRNA locus. LU snRNA is encoded within the second intron of *fas2*; *U5:23D* is located in the third intron. PSEA/PSEB, proximal sequence element A/B (see Figure S7 in Additional file 1 for alignment of the U11 and LU promoters in Drosophilids). Locations of a P-element insertion and two deficiencies are indicated. The arrows on the deficiencies indicate that the regions extend beyond the displayed area. **(b)** Complementation analysis of LU snRNA mutations and deficiencies. Numbers of third instar larvae are indicated in parentheses. **(c)** Localization of LU snRNA in S2 cells determined by *in situ* hybridization using LU sense and antisense probes. **(d)** Predicted secondary structure of *D. melanogaster* LU snRNA. **(e)** Phylogeny of LU snRNA. **(f)** Alignment of *Drosophilid* LU snRNA orthologs using LocARNA. The blue box indicates the Sm site. Half-brackets indicate covariant base pairs.

**Figure 6:**
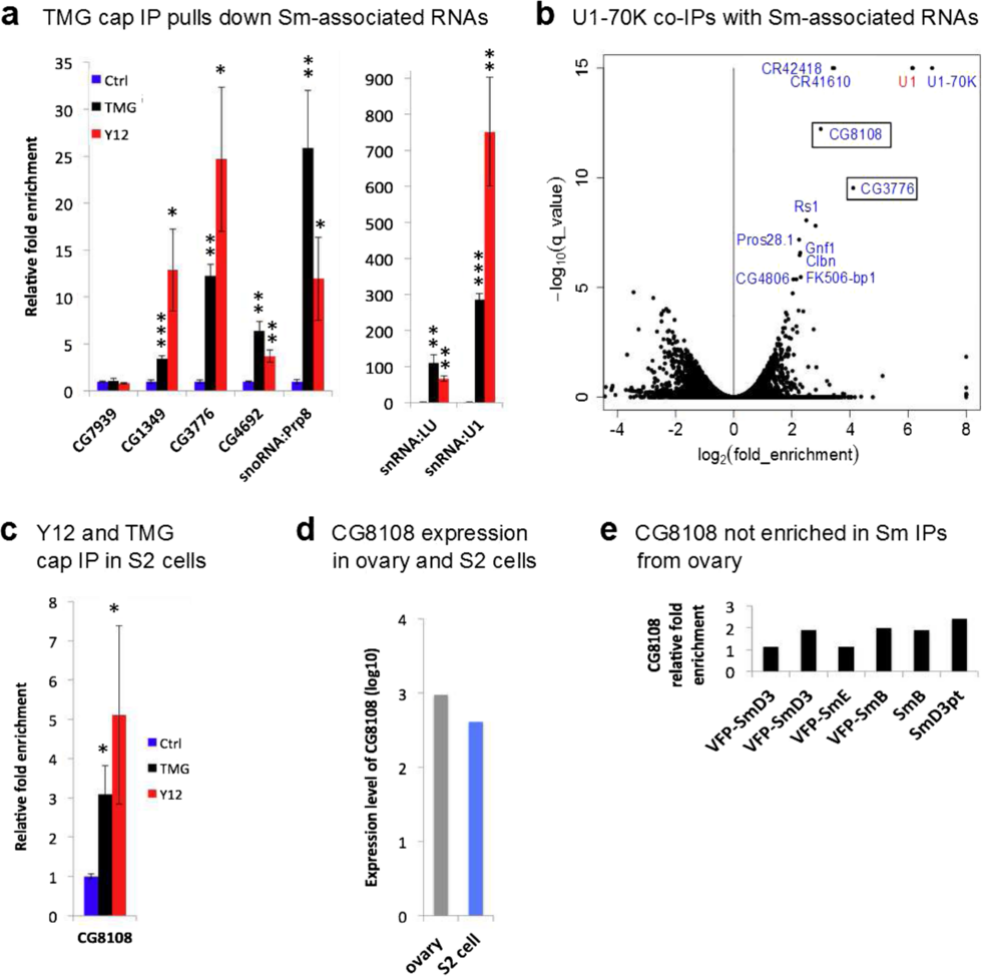
**snRNPs associate with mature mRNAs in S2 cells. (a)** Sm-associated mRNAs, as well as scaRNAs and snRNAs, can be pulled down by a TMG antibody in S2 cells. CG9042 (Gapdh) is used for normalization. **(b)** Enrichment analysis of the U1-70 K RIP-seq data in a volcano plot. The most highly enriched transcripts were labeled. The inset rectangular boxes highlight CG3776 and CG8108 mRNAs in the plot. Note: CG1349 and CG4692 could be associated with other snRNPs, and therefore not pulled down by U1-70 K. **(c)** CG8108 mRNA can be pulled down by TMG and Y12 antibodies in S2 cells. **(d)** CG8108 is expressed in similar levels in *Drosophila* ovary and S2 cells (data from FlyBase). **(e)** CG8108 mRNA is not enriched in ovary Sm RIP-seq. *t*-Test for significance between IP and control (Ctrl): *P < 0.05, **P < 0.01, ***P < 0.001). Error bars reflect the standard deviation.

We characterized the two Sm-associated ncRNAs and found that one, CR43708, has features typical of an snRNA. CR43708 is located in the second intron of *fas2* (CG3524, fatty acid synthase 2), a homolog of the human fatty acid synthase gene (Figure 5a). We defined the accurate 5′ and 3′ ends of CR43708, and found that this transcript is 116 nucleotides long (ZL and AGM, unpublished). Detailed analysis of sequences upstream of CR43708 revealed conserved proximal sequence elements PSEA and PSEB, highly similar to Sm-class snRNA promoters (Figure 5a; Figure S7a in Additional file 1) [47,48]. To examine the subcellular localization of CR43708, we carried out *in situ* hybridization in *Drosophila* S2 cells and found that this RNA accumulates in the nucleus (Figure 5c). Using the transcribed region and the promoter sequences, we searched genome and transcriptome databases for homologs. We recovered matches in nine species, all of which are in the melanogaster group of the *Drosophila* genus, and all are located within the same intron of the *fas2* gene (Figure 5e,f). Among the sequenced *Drosophila* species in the melanogaster group, the *Drosophila erecta* genome does not appear to contain CR43708, suggesting that it may have been lost. Interestingly, we found a truncated version of this gene within an intron of the *Ac3* gene in *D. melanogaster* (Figure S7c in Additional file 1). The homology extends through the first 70 bp of CR43708, and lacks the promoter and the 3′ end, suggesting that this paralog is a pseudogene. The predicted secondary structure of CR43708 closely resembles that of a canonical snRNA, including the presence of 5′ and 3′ end stem loops that flank a putative Sm binding site (Figure 5c). Structured sequence alignments clearly show that the putative Sm binding site (except in *Drosophila kikkawai*) and the terminal stem loops are well conserved. In addition, we identified many covariant base pairs within the two stem loops, supporting the predicted secondary structure (Figure 5f). Uridine-rich, Sm-class snRNAs such as U1 and U2 are known to contain a trimethyl-guanosine (TMG) 5′ cap structure that is generated upon formation of the Sm core RNP [9]. As expected, CR43708 was efficiently immunoprecipitated by anti-TMG antibodies (Figure 6a). Taken together, these features led us to conclude that this transcript is a novel Sm-class snRNA, which we termed snRNA:*LU* (*Like U*).

Interestingly, the *U5:23D* snRNA gene is located near *LU*, within a neighboring intron of the *fas2* protein coding gene (Figure 5a). We were unable to deduce the precise origin of *LU*; however, its juxtaposition with *U5:23D* suggests that it could have evolved from a U5 gene duplication, followed by rapid divergence. Supporting this notion, the 3′ end stem-loops of the LU snRNA homologs are quite similar to those of U5 snRNAs (Figure S7 in Additional file 1), although there is a lack of overall sequence similarity between the two genes.

To study the function of LU snRNA, we first considered the possibility that it might base pair with other snRNAs, as we found a nearly invariant single-stranded region located in the middle of LU snRNA (Figure 5d,f). Notably, we identified extensive base complementarity between this region of LU and the 5′ end of U6 (Figure S7d in Additional file 1). This putative base-pairing suggests that LU may be involved in splicing regulation. We identified four independent transposon insertions in and around the *LU* gene locus (see Materials and methods), and we confirmed that one of these insertion lines, *fas2*^k05816^, disrupts expression of both the *fas2* host gene and the *LU* snRNA gene (Figure 5a; Figure S7e in Additional file 1). Although homozygotes die around eclosion; complementation analysis between *fas2*^k05816^ and two other deletion lines uncovering this region suggests that neither the *fas2* host gene nor the *LU* snRNA gene are required for organismal viability (Figure 5b). We conclude that, although it may well contribute to organismal fitness, *LU* is not an essential gene. This conclusion is supported by the independent loss of LU snRNA in *D. erecta*. Taken together, our RIP-seq analysis of Sm proteins reveals that a total of 11 distinct species of Sm-class snRNAs are present in *Drosophila*: U1, U2, U4, U5, U6, U7, U4atac, U6atac, U11, U12 and LU.

#### Sm proteins associate with evolutionarily conserved and rapidly evolving scaRNAs

**Figure 7:**
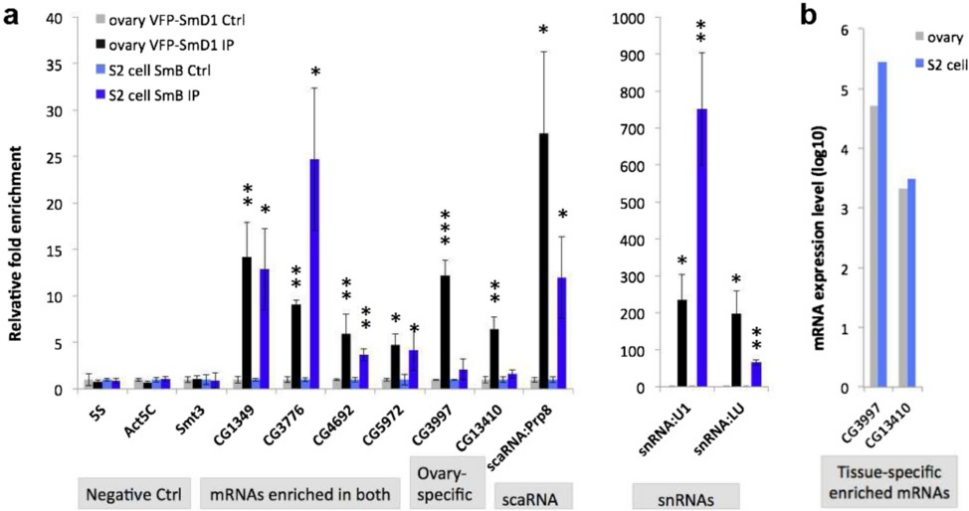
**RNA-Sm association is cell type-specific and not due to re-assortment. (a)** RIP-qRT-PCR in *da-Gal4 VFP-SmD1* fly ovary (anti-GFP) and S2 cells (Y12). Negative controls (Ctrl) used are 5S rRNA, Act5C and Smt3. CG9042 (Gapdh) is used as the normalization standard. snRNAs are shown separately due to the difference in scale. **(b)** mRNAs associated with Sm proteins in ovaries but not in S2 cells are expressed in S2 cells. *t*-Test for significance between IP and Ctrl: *P < 0.05, **P < 0.01, ***P < 0.001. Error bars show standard deviation.

scaRNAs are ncRNAs that guide methylation and pseudouridylation of snRNAs, the specificity of which is determined by base-pairing with targets [49]. A previous study showed that in human cells, several scaRNAs specifically associate with SmB and SmD3, including U85, U87, U89 and human telomerase RNA (hTR) [50]. Co-precipitation of SmB/D3 with these scaRNAs was shown to require the conserved CAB box [50], which is essential for scaRNA localization to Cajal bodies [51]. To determine whether other ncRNAs co-purify with Sm proteins in *Drosophila* and human cells, we systematically analyzed the enrichment values of snoRNAs and scaRNAs in our RIP-seq datasets. Consistent with the findings of Fu and Collins [50], we found that two previously identified *Drosophila* scaRNAs, U85 (CR32863 or snoRNA:MeU5-C46) and CR33716 (snoRNA:MeU5:U42), were enriched in the Sm protein IPs (Figure 4a; Table S5 in Additional file 1). Interestingly, the new Sm-associated ncRNA identified in this study (CR43600 or snoRNA:Prp8) also appears to have features of box H/ACA scaRNAs. Indeed, evolutionary comparisons identify conserved H/ACA and CAB box elements present within the detected orthologs (Figure S6b,c in Additional file 1). snoRNA:Prp8 folds into a predicted secondary structure similar to that of other box H/ACA scaRNAs, which is further supported by the presence of multiple covariant base pairs. In support of the notion that snoRNA:Prp8 is an H/ACA box scaRNA, we searched snRNAs for sequence complementarity to the pseudouridylation pocket sequences, and found potential target sites in U1, U5, U7 and U11 (Figure S6d in Additional file 1). Therefore, we have renamed this transcript scaRNA:Prp8. We detected homologs of scaRNA:Prp8 in both Diptera (Drosophilids, *Anopheles gambiae*) and Hymenoptera (*Apis mellifera*), but not in Coleoptera (*Tribolium castaneum*) (Figure S6b in Additional file 1). The orthologous scaRNA:Prp8 RNAs are highly conserved, suggesting their functional importance. Many scaRNA and snoRNA genes reside within introns of splicing and translation-related genes, respectively [52]. The nested gene structures are thought to facilitate transcriptional co-regulation. Thus, it is not surprising that the *Prp8* host gene encodes a splicing factor (Figure S6a in Additional file 1) [53,54]. Although Fu and Collins [50] reported that only SmB and SmD3 co-purified with scaRNAs such as hTR, we found that IP targeting VFP-SmD1 also pulled down snoRNA:Prp8 (Figure 7a). It has been shown that many H/ACA box scaRNAs are TMG-capped [55-58]; consistent with these studies, we also found that scaRNA:Prp8 co-immunoprecipitates with anti-TMG antibodies (Figure 6a).

To identify additional Sm-associated ncRNAs in HeLa cells, we examined known human sno/scaRNA loci. Several of the previously reported scaRNAs, including U85, U87 and U89, showed moderate but significant enrichment in Y12 IPs (Figure 4b; Table S7 in Additional file 1). In addition, we found several other scaRNAs that are highly enriched (Figure 4b; Table S7 in Additional file 1). However, we did not detect any significant enrichment of hTR as previously reported [50] (data not shown). We identified a novel, unannotated Sm-associated ncRNA, which we named SHAN (Sm-associated Hybrid tRNA^Asp^-containing NcRNA); its predicted secondary structure is shown in Figure S8c in Additional file 1. This new transcript appears to be a chimera between a tRNA gene and an H/ACA type scaRNA gene. Supporting this hypothesis, we detected H box, ACA box and CAB box motifs in the orthologous sequences from other primates (Figure S8b,c in Additional file 1). In summary, our RIP-seq analysis revealed both evolutionarily conserved and newly evolved interactions between Sm proteins and scaRNAs, suggesting that Sm proteins play roles in the biogenesis/function of a subset of scaRNAs. However, we did not identify sequence/structural features that distinguish Sm-associated scaRNAs from other scaRNAs.

#### Sm proteins associate with mRNAs encoding mitochondrial and translation-related proteins

Due to a relative lack of comprehensive annotation of *Drosophila* gene ontology, we manually annotated the Sm-associated mRNAs by homolog searching, protein domain analysis, and literature mining. This analysis surprisingly revealed two major categories of mRNAs: those encoding ribosome/translation-related proteins (13/86), and mitochondrial proteins (including mitochondrial ribosomal proteins, 19/86). As discussed above, the enrichment of ribosomal protein mRNAs is not simply due to high levels of expression. Only a subset of ribosomal protein mRNAs is enriched in the Sm protein IPs. For example, mRNAs encoding RpS11 (CG8857) and RpL39 (CG3997) are highly enriched in Sm protein IPs (Figure 3a; Table S5 in Additional file 1), whereas RpL19 (CG2746) and RpL4 (CG5502) are not enriched at all (Figure 4a and data not shown). Anecdotally, the mRNA encoded by CG3776, which is highly enriched, is located immediately adjacent to RpL19 in the *Drosophila* genome, demonstrating the high degree of specificity of our approach.

Two other *Drosophila* Sm-associated mRNAs merit special interest. CG4692 encodes a predicted mitochondrial F_1_-F_O_ ATP synthase subunit that was consistently enriched in our IPs. We found that this mRNA localizes to the actin-rich oocyte cortex of late-stage *Drosophila* egg chambers (Figure S4 in Additional file 1), in a pattern that is very similar to that of VFP-tagged Sm proteins, as described previously [21]. Analysis of several other high-scoring mRNAs from Figure 3a and Figure S4 in Additional file 1 did not display this pattern (data not shown), so it is not a general feature of Sm-associated mRNAs, but was nonetheless interesting. CG1349 (dj-1beta) encodes a *Drosophila* homolog of the human DJ-1/PARK7 (Parkinson autosomal recessive, early onset 7) gene. DJ-1/PARK7 is one of 10 genes identified to date that cause familial Parkinson disease [59]. A subpopulation of DJ-1 protein is localized to mitochondria in a regulated manner, and is required for proper mitochondrial function [60]. Thus, it is possible that Sm proteins play a role in regulating the localization and/or translation of associated mRNAs.

In contrast to the more than 70 Sm-associated mRNAs in the fruitfly (Figure 3a), we identified roughly 30 high-scoring mRNAs in human cells (Figure 3b). The lower number in the human dataset is potentially due to a reduced coverage of the transcriptome. Nevertheless, we found that one of the replication-dependent histone mRNAs, HIST2H2AB, is highly enriched in the IPs (Figures 3b and 4b). In contrast, two adjacent histone genes, HIST2H2BE and HIST2H2AC, were not enriched (Figure 4b). Another histone mRNA (HIST1H2AM), was also significantly enriched (Figure 3b). Interestingly, Steitz and colleagues [34] previously showed that the U2 snRNP binds to (intronless) histone pre-mRNAs and stimulates 3′ end processing. Our identification of histone mRNAs in Sm protein co-IPs may reflect a snRNP-mediated interaction between Sm proteins and mRNAs. However, none of the *Drosophila* replication-dependent histone mRNAs were enriched in the Sm protein IPs (Figure S10 in Additional file 1). Taken together, our data suggest that the mode of interaction between Sm proteins, snRNPs and mRNAs is conserved between vertebrates and invertebrates.

### Validation and tissue-specificity of RNA-Sm protein interactions in *Drosophila*

We have shown that the B/D3 and E/F/G subcomplexes bind essentially the same set of target RNAs. To determine whether SmD1 (which forms heterodimers with SmD2; Figure 1b) also associates with the RNAs listed in Figure 3a, we immunopurified ovarian RNA from *daGal4, VFP-SmD1* flies (using anti-GFP) and carried out qRT-PCR. Furthermore, to assay the observed interactions in another cell type, we also performed qRT-PCR on RNAs immunopurified from S2 cells using anti-Sm antibody Y12. We chose six of the top-ranking mRNAs that were identified in the RIP-seq experiments (targeting SmB, SmD3 and SmE), and found that they were all highly enriched in the VFP-SmD1 IPs (Figure 7a). Two snRNAs (U1 and LU) were used as positive controls, whereas three RNAs not expected to interact with Sm proteins (Act5C and Smt3 mRNAs and 5S rRNA) were used as negative controls (Figure 7a). In contrast to the results in ovaries, only four out of the six mRNAs we tested were significantly enriched in the S2 cell IPs (Figure 7a). Given that the Sm proteins and the six mRNAs we tested all have comparable expression levels in both ovaries and S2 cells (Figure 7b and data not shown), these findings suggest that the interactions between mRNAs and Sm proteins can be tissue-specific. A potential concern in all RIP experiments is that the co-purification of the components might be due to reassortment of complexes following cell lysis [61,62]. However, the fact that CG3997 and CG13410 fail to associate with Sm proteins despite the fact that they are well expressed in S2 cells argues strongly against this artifact.

### Sm proteins associate with fully spliced and polyadenylated mRNAs

**Figure 8:**
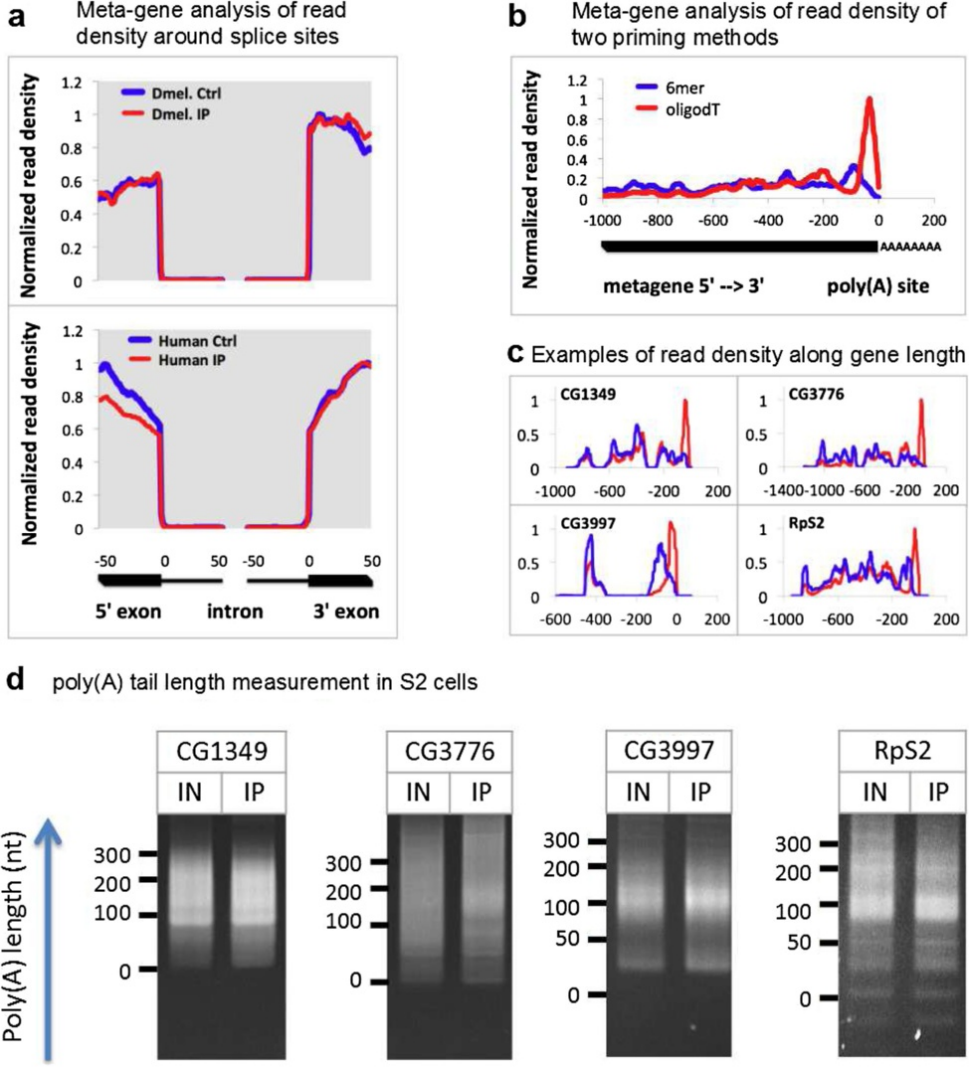
**Sm proteins associate with mature mRNAs. (a)** Meta-gene analysis of read density around splice sites for all *Drosophila* and human Sm-associated intron-containing mRNAs in all RIP-seq experiments. **(b)** Meta-gene analysis of read density along the gene length for all *Drosophila* Sm-associated mRNAs quantified from oligodT and random hexamer primed libraries. **(c)** Example tracks for read density along the gene length for oligodT and random hexamer primed libraries. **(d)** Poly(A) tail length Sm-associated mRNAs (CG3997, CG1349 and CG3776) and non-associated mRNA (RpS2) from Y12 IP in S2 cells. IN, input total RNA; IP, immunoprecipitated RNA. The labels denote the length of poly(A) tails. Oligo(dT)_20_ was used as the reverse primer for the reverse transcription and subsequent PCR, therefore producing the ‘smear’ of poly(A) tail. See Figure S11 in Additional file 1 for analysis of poly(A) containing reads for selected Sm-associated mRNAs.

The identification of significantly enriched mRNAs in the co-IP fractions led us to ask whether the association between Sm proteins and mRNAs was due to the splicing reaction itself. In other words, do Sm proteins interact with partially spliced or fully mature mRNAs? A quick glance at Figure 3 shows that the read depth over intronic sequences is very low. Meta-gene analysis of both *Drosophila* and human Sm-associated intron-containing mRNAs showed that the vast majority of reads map to exons, and the IPs did not pull down more pre-mRNAs than the controls did (Figure 8a). Among the few transcripts that showed significant numbers of intronic reads, most of those were actually candidates for either new exons or new genes (for example, scaRNA:Prp8 and snRNA:LU; Figure 4a). Thus, this analysis demonstrates that the mRNAs that associate with canonical Sm proteins are fully spliced. Importantly, 6 of the 72 *Drosophila* Sm-associated mRNAs (CG6008, CG13151, CG13951, CG17531, CG11076 and CG7137), and 2 of the 30 human Sm-associated mRNAs (HIST2H2AB and HIST2H2AM) are intronless, suggesting that splicing is not a prerequisite for Sm protein interaction.

The highly conserved eukaryotic Lsm1-7 complex is known to bind to mRNA degradation intermediates, preferentially those with oligoadenylated tails [14,63]. We therefore asked whether the canonical Sm ring shares this same recognition specificity. Taking advantage of the oligo(dT)_20_ and random hexamer primed RIP-seq cDNA libraries, we compared the read coverage patterns for the various mRNAs. As shown in Figure 8b,c, there is a dramatic 3′ end bias in the oligo(dT)_20_ primed libraries compared to the randomly primed ones. We also confirmed the presence of adenylated tails of Sm-associated and non-associated mRNAs by examining the unmappable reads in the oligo(dT)_20_ primed RIP-seq files (Figure S11 in Additional file 1). In order to measure polyA tail lengths, we performed RACE-PAT (rapid amplification of cDNA ends-poly(A) tail assay) on immunopurified RNAs from S2 cells [64]. This analysis demonstrates that the poly(A) tails of the Sm-associated mRNAs are roughly the same length as the input mRNAs (Figure 8d). Taken together, these data show that Sm and Lsm proteins have distinct specificities and modes of mRNA interaction.

### Sm protein interaction with mRNAs is mediated by snRNPs

The association of snRNAs and scaRNAs with Sm proteins is thought to be mediated by direct binding to Sm sites and CAB boxes, respectively [50,65,66]. We therefore wanted to determine whether Sm proteins associate with mRNAs directly or indirectly. Toward that end, we carried out PAR-CLIP (photoactivatable ribonucleoside-enhanced crosslinking and immunoprecipitation) on native and VFP-tagged Sm complexes [67]; however, we were unable to detect any significant crosslinking events in the precipitated RNA (data not shown). We note that canonical Sm proteins are notoriously poor at crosslinking. Even on extremely abundant targets such as U1 snRNA, the UV crosslinking efficiency was rather low, with SmG being the predominant crosslinked member of the heptameric ring [68]. More recently, Castello *et al*. [69] carried out UV- and PAR-CLIP in parallel to generate a comprehensive mRNA interactome in HeLa cells. As part of their studies, they identified the Lsm1-7 proteins as mRNA binding proteins, but the canonical Sm proteins were not detected, again supporting the idea that Sm proteins are not efficiently crosslinked to mRNAs.

However, the fact that we found all three Sm sub-complexes in association with the same set of mRNAs (Figures 2 and 3) suggested interaction with a complex that contains an intact Sm ring. Furthermore, the previously reported binding between histone mRNAs and U2 snRNPs [34], coupled with our identification of H2A mRNAs in our RIP-seq data (Figure 4) led us to ask whether the mRNA-Sm interaction might be indirect, mediated by snRNPs. Sm-class spliceosomal snRNAs are transcribed by a specialized form of RNA polymerase II and contain a 5′ TMG cap structure [9]. Using anti-TMG antibodies, we immunopurified RNPs from S2 cell lysate and used qRT-PCR to assess the enrichment of mRNAs. As expected, the U1 and LU snRNAs (positive controls) were highly enriched in the anti-TMG IPs, whereas CG7939 (RpL32) mRNA was not (Figure 6a). Notably, the scaRNA:Prp8 transcript and all three of the Sm-associated mRNAs we tested (CG1349, CG3776 and CG4692) were significantly enriched in the anti-TMG pulldowns (Figure 6a). In parallel, we performed anti-TMG IPs using purified S2 cell RNA (that is, the IP was not performed in lysates). We detected significant enrichment of U1 snRNA but not the mRNAs (Figure S12 in Additional file 1). Therefore, the Sm-associated mRNP complex contains a TMG cap component that is structurally distinct from the mRNAs themselves, suggesting the presence of snRNPs.

In order to test whether the interactions with mRNAs are indirectly mediated by snRNPs, we took advantage of a database from a large-scale *Drosophila* S2 cell RIP-seq analysis of 29 RNA binding proteins, including U1-70 K [70]. The U1-70 K protein binds to U1 snRNA directly and specifically, thus allowing it to be used as an additional, independent epitope for pulldown experiments [68]. We mined the database for RNAs that associate with U1-70 K by analyzing RNAs that were enriched in IPs from U1-70 K transfected versus non-transfected cells. The RIP-seq data were displayed on a volcano plot to identify transcripts that are highly enriched in the IPs. As shown in Figure 6b, U1 snRNA, but not the other spliceosomal snRNAs, was dramatically enriched in the IP fractions, along with a number of other ncRNAs and mRNAs. Among this latter category, three mRNAs were particularly noteworthy: CG3776, CG8108 and U1-70 K (CG8749) itself. Although U1-70 K protein may well bind to its own mRNA for some type of autologous feedback, one must view this result with caution because the cells were transiently transfected with U1-70 K cDNAs, artificially inflating expression of this transcript. However, CG3776 and CG8108 remain good candidates. Interestingly, CG3776 was one of the top-ranking candidates in our ovarian RIP-seq experiments (Figures 3 and 4), but CG8108 was not identified as being enriched, even though it is expressed at similar levels in S2 cells (Figure 6d,e). Because the U1-70 K data were generated from S2 cells, we performed anti-TMG and anti-SmB (Y12) IPs in S2 cells, followed by qRT-PCR. As shown in Figure 6c, we detected significant enrichment of CG8108 in both the TMG and Sm protein IPs. These data provide additional support for the idea that the Sm-mRNA interactions are cell-type specific and not due to reassortment, as CG8108 is expressed in *Drosophila* ovaries (Figure 6d) but not significantly enriched in Sm protein IPs (Figure 6e).

**Figure 9:**
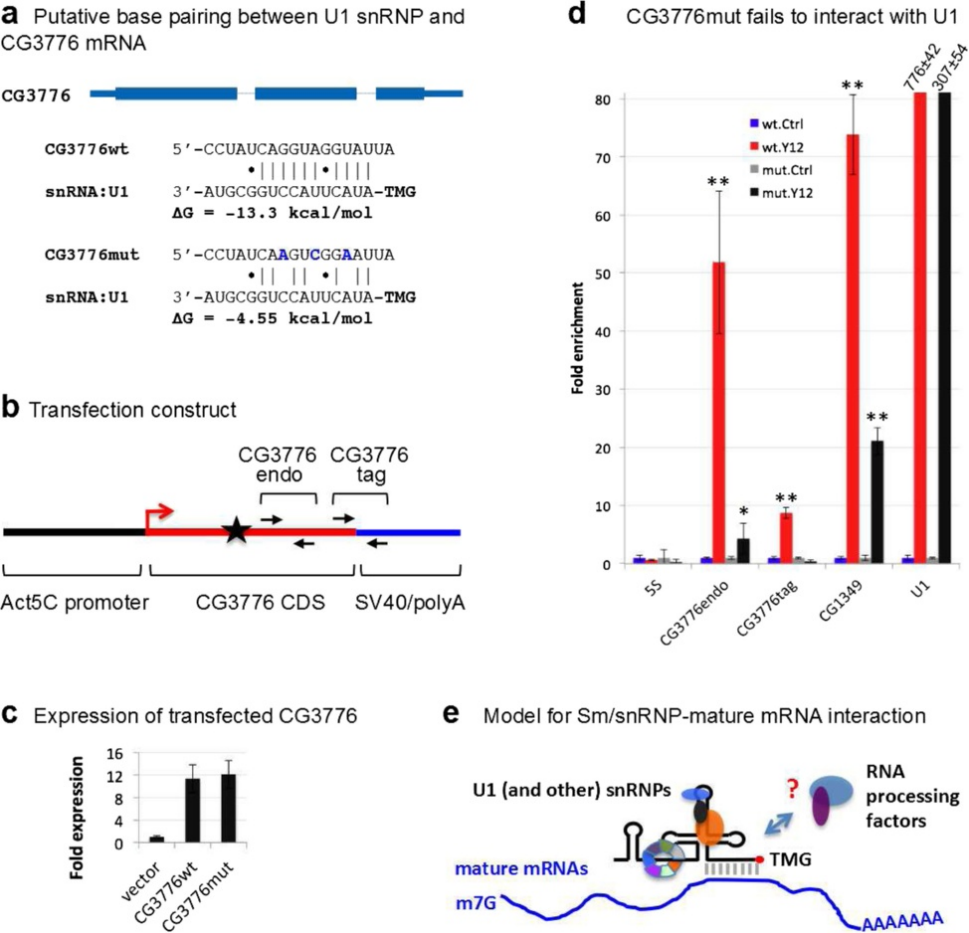
**U1 snRNP binds mature mRNAs. (a)** Putative base pairs between the 5′ end of U1 snRNA and the CG3776 mRNA coding region (upper panel). Within the putative region of base pairing, three translationally silent point mutations were introduced (bold blue letters) to disrupt the helix (lower panel). **(b)** Cartoon of the S2 cell transfection construct, showing the CG3776 expression unit. CG3776endo and CG3776tag indicate locations of primers for qRT-PCR. CG3776endo amplifies both endogenous and transfected CG3776 mRNAs, whereas CG3776tag amplifies transfected CG3776 mRNA only. The black star indicates the location of the putative U1 binding site. **(c)** pAW vector, pAW-CG3776wt and pAW-CG3776mut were transfected into S2 cells, and CG3776wt and CG3776mut expression was measured using qRT-PCR with the CG3776endo primer pair. GAPDH was used as normalization standard. **(d)** After pAW-CG3776wt and pAW-CG3776mut were transfected, anti-Sm (Y12) IPs were performed using S2 cell lysate. GAPDH was used as normalization standard. **(e)** Proposed model of snRNP-mRNA interactions. Distinct snRNPs (U1 and potentially others) associate with mature mRNAs via base pairing and/or protein-mediated interaction. Such interactions could serve as a platform to recruit RNA processing factors that act on multiple levels of RNA metabolism. *t*-Test for significance between IP and control (Ctrl): *P < 0.05, **P < 0.01, ***P < 0.001. Mut, mutant; wt, wild-type.

In addition to CG3776, we also found other U1-70 K associated RNAs that overlapped with our Sm protein dataset, including CG5972 and CR32863. Although it is likely that U1-70 K binds to certain RNAs in a manner that is independent of the U1 snRNP, the overlap between our anti-Sm and anti-TMG data suggests that a cadre of mature mRNAs interacts with intact snRNPs outside of the spliceosome. Thus, we checked for sequence complementarity in CG3776 mRNA and found a 12 bp perfect duplex with the 5′ end of U1 snRNA (Figure 9a). The complementary region is in the middle of the second exon of CG3776, far from any intron-exon boundaries and the base-pairing potential is much greater than is typical for a 5′ splice site. Similarly, we found stretches of complementarity between U1 snRNA and exonic regions of CG8108, CG5972 and many other transcripts (Figure S13 in Additional file 1). Those mRNAs within our dataset that are missing from the U1-70 K pulldowns (for example, CG1349 and CG4692) are plausibly bound by other Sm snRNPs such as U2, U4/U6, U5, U11 and U12. A list of such potential base pairing interactions was compiled by taking known single-stranded regions from snRNAs, and using them to find putative binding sites on the list of Sm- and U1-70 K-associated mature mRNAs (Figure S13 in Additional file 1). We found many potential sites with a duplex length and minimum free energy profile similar to the ones shown in Figure 6f. Taken together with the Sm and TMG IPs, these data suggest that snRNPs associate with subsets of mature *Drosophila* mRNAs, in a mode that is distinct from their interactions within the spliceosome.

To test whether base pairing between U1 snRNP and CG3776 mRNA is responsible for their interaction, we introduced three synonymous point mutations within the twelve-nucleotide complementary region in CG3776 mRNA that should completely block putative pairing with U1 snRNA (Figure 9a). We then transfected both wild-type and mutant CG3776 mRNA expression constructs into S2 cells (Figure 9b). The constructs are transcribed by an *Act5C* promoter and are terminated using the SV40 polyA signal and a heterologous 3′ UTR. We confirmed that both transfections produced similar levels of chimeric CG3776 mRNAs (Figure 9c) and then performed Y12 IPs on S2 cell lysates, using normal goat serum as a control. As expected, 5S rRNA was not enriched in the IP fractions, whereas CG1349 mRNA and U1 snRNA were both significantly enriched in the transfections. Both endogenous and transfected CG3776wt mRNAs were pulled down by the Y12 antibody, whereas transfected CG3776mut mRNA was not (Figure 9d). These results support two conclusions. First, splicing is not required for U1 snRNP binding, and the binding site for U1 snRNP is located within the CG3776 mRNA coding sequence, since it can be efficiently pulled down by Y12 antibody. Second, the predicted U1 binding site is indeed necessary for U1 snRNP binding. Taken together, our results suggest that snRNPs bind mature mRNAs, and that at least one mechanism requires U1 snRNP base pairing with target mRNAs.

## Discussion

We have developed an experimental and analytical pipeline to identify RNAs that stably associate with Sm proteins, an evolutionarily ancient group of RNA binding factors. The targeting of multiple subunits of an RNA-binding complex in this RIP-seq approach, along with the use of different genetic backgrounds, ensures that the identified RNPs are *bona fide*. Notably, this pipeline can be easily adapted to study other RNA-binding complexes.

### Sm proteins in scaRNP complexes

We found that subsets of scaRNAs associate with Sm proteins, in both *Drosophila* and human cells. These include the highly conserved U85 scaRNA and newly evolved and non-canonical scaRNAs, such as scaRNA:Prp8 and SHAN, identified in this study. The involvement of Sm proteins in scaRNP biogenesis and function has been shown in several previous studies. Notably, both budding and fission yeast telomerase RNA precursors contain canonical Sm sites and are directly bound by Sm proteins [56,71]. In fission yeast, Sm binding to telomerase RNA stimulates spliceosome-mediated cleavage that mimics the first step of splicing [57,72]. However, none of the scaRNAs we found in our IPs contain readily identifiable Sm sites. Fu and Collins [50] reported that SmB and SmD3, but not other Sm proteins, specifically associate with several human scaRNAs, and that this association requires a conserved CAB box sequence. Tycowski *et al*. [73] showed that this CAB box is bound by a protein called WDR79. In our comprehensive analysis of fruit fly and human Sm-associated scaRNAs, we did not find additional sequence or structural features that distinguish them. Thus, these studies suggest an evolutionarily conserved role for Sm proteins in scaRNA biogenesis and function; however, the mechanism through which scaRNAs that lack identifiable Sm sites associate with Sm proteins is not well understood.

### Splicing-independent, evolutionarily ancient functions for Sm-class snRNPs

The available single-stranded regions of snRNPs, which are used to identify intron-exon boundaries and intronic splicing elements, also serve as prime candidates for base pairing with mature mRNAs. We propose a model whereby Sm-class snRNPs interact with their targets via a combination of base pairing and protein-RNA interactions, as shown in Figure 9e. Indeed, this model has precedence, as the efficacy of this combination of interactions has already been demonstrated. Steitz and colleagues [34] showed that both RNA-RNA and protein-RNA interactions are individually sufficient for function of the SF3b-hPrp43 subcomplex within the U2 snRNP in stimulating histone mRNA 3′-end maturation. In the current study, we showed that a sequence within CG3776 mRNA that potentially base pairs with the 5′ end of U1 snRNP is required for binding. Mutation of this sequence abrogates U1 binding. By such a mechanism, snRNAs and/or specific proteins that bind to snRNPs could recruit other factors that, together, serve to regulate the processing, localization, translation or degradation of target mRNAs (Figure 9e).

Recently, Berg *et al*. [12] proposed a function for U1 snRNPs, termed ‘telescripting,’ whereby binding of U1 to nascent transcripts acts to suppress premature cleavage and polyadenylation at cryptic sites. Reduction of U1 snRNP levels elicited shortening of 3′ UTR length and proximal 3′ exon switching of numerous transcripts in a dose-dependent fashion [11,12]. This process is distinct from the interactions described here, as our data clearly showed snRNPs associating with mature mRNAs. Moreover, we did not observe significant enrichment of intronic regions in our RIP-seq datasets, as might have been expected if the telescripting interactions between U1 and post-splicing lariats were stable. Thus, the interactions described here with mature mRNAs are stable, likely taking place either in the cytoplasm or just prior to mRNA export.

Furthermore, the data indicate that U1 snRNP is not the only Sm RNP that associates with mature mRNAs. The U2 snRNP-histone mRNA interaction [34] (and this work) is a case in point. We did not detect any downstream flanking sequences in our RIP-seq data, suggesting that the U2 snRNP maintains contact with the histone mRNA long after 3′ end maturation, and therefore a potential function downstream of 3′ end formation, for example, translational control. We also identified Sm- and TMG-associated mRNAs in S2 cells that are not enriched in U1-70 K IPs, most prominently CG1349 and CG4692. Interestingly, we found that the localization pattern of *Drosophila* CG4692 within stage 10 egg chambers (Figure S9 in Additional file 1) mirrored that of VFP-tagged Sm proteins [21]. Taken together, these findings suggest a general role for Sm-class snRNPs in post-splicing mRNA metabolism.

The Sm family of proteins is evolutionarily ancient. The eukaryotic Lsm1-7 complex regulates mRNA decapping and degradation by association with oligoadenylated mRNAs [15,74,75]. The bacterial Sm orthologue, Hfq, also functions to regulate the translation and stability of a number of transcripts (for review see [76]). Similar to eukaryotic Sm proteins, prokaryotic Hfq forms a toroidal ring that binds a class of 50- to 200-nucleotide small (s)RNAs. These so-called ‘sRNPs’ bind to their targets, which include ribosomal protein (RP) mRNAs, via a combination of base pairing and protein-RNA interactions [6,7,76-79]. Although the RP genes are not homologs of the RP mRNAs identified in this study, our findings nevertheless support the hypothesis that regulation of ribosome biogenesis is a deeply conserved function of Sm proteins.

Sequence covariation is generally considered a hallmark of conserved base-pairing interactions, underscoring functional importance. Not surprisingly, we found many covariant base pairs in the stem-loops of snRNA:LU and scaRNA:Prp8, despite their short evolutionary histories (Figure 5; Figures S6 and S7 in Additional file 1). However, we were unable to analyze this feature in our *Drosophila* and human Sm/snRNP-associated mRNAs, as no clearly orthologous mRNA transcripts were identified. Instead, we found that most of the targets of Sm proteins and snRNPs are different in the flies and human, with the exception of snRNAs and U85 scaRNA. This is consistent with the idea that protein-RNA and RNA-RNA interaction networks rapidly rewire themselves during evolution, despite the conservation of the individual components. For example, several studies on the RNA targets of Puf family proteins in yeast, fruit fly and human suggest that even though the binding sites of the proteins are conserved, the target mRNAs are not [41,80,81]. Similarly, Graveley and colleagues [82] showed that the binding sites for PS and NOVA1/2 are highly conserved between insects and mammals, but the target gene orthologs associated with PS and NOVA1/2 are almost entirely non-overlapping. This change of regulatory relationships in evolution has also been observed in the processing of minor introns and highly conserved microRNAs, such as let-7 and its targets [83,84].

### Technical considerations

It is likely that the Sm-associated transcriptome is larger than the one described here. Although RNA-seq is quite sensitive, it may not be sensitive enough to reliably identify all of the low abundance transcripts from the relatively minute amount of immunopurified RNAs. The spliceosomal snRNAs comprise a majority of the immunopurified transcripts, limiting the ability of the sequencer to identify low abundance Sm-associated RNAs, especially scaRNAs and mRNAs. In addition, we employed a very stringent analysis procedure to ensure that the identified targets were not false positives. This procedure could also lead to false negatives. In our normalization, we assumed that the majority of RNAs do not associate with Sm proteins. This may or may not be true. There could be a very large number of transcripts that associate with Sm proteins with lower affinities than the ones identified in this study. The extent to which our assumption holds true will dictate the number of false negatives. Finally, as our qRT-PCR results suggest, certain RNA targets associate with Sm proteins in a tissue-specific fashion. Therefore, a comprehensive RIP-seq analysis of different tissues would be needed in order to identify all the targets of Sm proteins.

Recently, RNA crosslinking has been extensively used in characterizing targets of RNA binding proteins [66-68,85,86]. These methods not only provide evidence for direct interaction between RNAs and proteins, but can also achieve single-nucleotide resolution of the binding sites. However, such methods are not applicable to complexes that are refractory to crosslinking or interactions that are indirect. Canonical Sm proteins are poor substrates for UV crosslinking, even to the highly abundant snRNAs [66,68]. A more recent study used two different crosslinking methods to characterize the mRNA-associated proteome; they also failed to detect the canonical Sm proteins [69]. These investigators also identified the eIF4AIII component of the exon-junction complex (EJC), but not the other three EJC subunits [69], which are presumably beyond the effective crosslinking radius. Because only eIF4AIII makes a direct contact with the mRNA, this result further supports the notion that crosslinking is not effective for studying all RNA-protein interactions. Our multiple-targeting strategy is therefore advantageous for the study of multimeric RNP complexes. The use of mock IPs as controls enables direct quantification of enrichment ratios, providing valuable information about the stability and affinity of the protein-RNA complexes. This point is illustrated by our RIP-seq data: the direct snRNA-Sm protein interactions are very stable, and correspondingly have much higher enrichment ratios than the mRNAs, which associate with Sm proteins indirectly.

## Conclusions

The structural and functional similarities between prokaryotic sRNPs and eukaryotic snRNPs suggest that canonical Sm-class snRNPs have the potential to carry out multiple functions inside the eukaryotic cell. This study represents the first comprehensive analysis of eukaryotic Sm-containing RNPs, and provides a basis for additional functional analyses of Sm proteins/snRNPs outside of the context of pre-mRNA splicing. We have developed a flexible experimental procedure and robust statistical analysis methods to identify mRNAs that are associated with canonical Sm proteins in *Drosophila* and human cells. Using this pipeline, we confirmed and extended previous reports that Sm proteins associate with snRNAs, scaRNAs and histone mRNAs. Importantly, we also identified numerous Sm-associated mRNAs, along with several novel, previously unannotated snRNA and scaRNA transcripts. These newly discovered snRNAs and scaRNAs are highly conserved in the species with detectable homologs, suggesting that they are functionally important. The evidence indicates that the mRNA-Sm protein interaction is neither a consequence of splicing nor a product of Lsm1-7-dependent mRNA degradation. Instead, the interactions are mediated by snRNPs with mature mRNAs. Moreover, the fact that we did not identify intron-retained pre-mRNAs strongly suggests that the association between Sm proteins/snRNPs and mature mRNAs is more stable than the interactions within the spliceosome.

## Materials and methods

### Fly strains and cell lines

These previously described fly strains were used: Oregon R (OR, as the wild type), *nos-Gal4 VFP-SmB*, *nos-Gal4 VFP-SmD3*, *nos-Gal4 VFP-SmE*, *da-Gal4 VFP-SmD1*, *SmD3pt* and *Tralpt* from the fly-trap project [21,87,88]. We characterized the insertion sites of P elements around the *LU* gene, and they are listed as follows. Line 10580 (k05816, y^1^ w^67c23^; P{lacW}v(2)k05816^k05816^, l(2)k05816^k05816^/CyO, from Bloomington Stock Center) and line 111186 (k05816, y^d2^ w^1118^ P{ey-FLP.N}2 P{GMR-lacZ.C(38.1)}TPN1; P{lacW}v(2)k05816^k05816^ P{neoFRT}40A/CyO y^+^, from DGRC, Kyoto): CCCATCGAGT|GTCGGGGATC; line d04154 (P{XP}v(2)k05816^d04154^): TCATAGCAAA|CATCCACCCC; line 203640 (y^1^ w^67c23^; P{GSV7}GS22096/SM1, from DGRC, Kyoto): CGGCGCAAGT|GGCTGACTCA; line 103535 (y* w*; P{GawB}v(2)k05816^NP0131^/CyO, P{UAS-lacZ.UW14}UW14, from DGRC, Kyoto):CAACTGGTTA|TGGCAAGCCA. The following deficiency lines were obtained from stock collections: Df(2 L)Exel7014/CyO (Exelixis collection at Harvard, stock no. 7784), and Df(2 L)BSC162/CyO (BDSC at Bloomington, stock no. 9597). The flies were cultured on standard corn meal food at room temperature (22°C) with 12 hour light-12 hour darkness cycles. *Drosophila* S2 cells were cultured in Express Five (Life Technologies, Carlsbad, CA, USA) plus 10% fetal bovine serum and penicillin/streptomycin, at room temperature (22°C). Human HeLa cells were cultured in DMEM (Life Technologies) plus 10% fetal bovine serum and penicillin/streptomycin, in a 37°C incubator with 5% CO_2_.

### RIP-seq experiment

#### Drosophila *ovary RIP-seq*

These antibodies were used for IPs: Y12 (J Steitz, Yale, New Haven, CT, USA) [89], rabbit anti-GFP antibody (Abcam, ab6556, Cambridge, UK), agarose-conjugated anti-TMG (Calbiochem, La Jolla, CA, USA). For the *Drosophila* RIP-seq, ovaries were dissected from well-fed 3- to 4-day-old female flies. The IPs, RNA purification and reverse transcription were done essentially as described [21]. After first strand synthesis, the second strand was made using RNase H and DNA polymerase I (Life Technologies, Carlsbad, CA, USA) according to the manufacturers’ instructions. The resultant double-stranded cDNA was fragmented, ligated with Illumina sequencing adapters and sequenced in 36 cycles using the Genome Analyzer II platform at the UNC High Throughput Sequencing Facility. Random hexamer priming was used for reverse transcription for all seven cDNA libraries. In parallel, we also used oligo(dT)_20_ priming to generate cDNA libraries for four of the seven samples (Table S1 in Additional file 1).

#### Human HeLa cell RIP-seq

HeLa cells were lysed and immunoprecipitated using the Y12 antibody. Four IPs and four normal goat serum controls (mock IP) were performed at the same time. The cDNA from these four controls and four IPs was used for real-time PCR analysis of selected transcripts. The RNA from two controls and two IPs was converted to cDNA libraries according to the Illumina TruSeq RNA SamplePrep Guide (version 2). The HeLa cell RIP-seq libraries were sequenced in 50 cycles.

The RIP experiments for qRT-PCR were performed under more stringent conditions: 150 mM NaCl, 0.5% NP-40, 50 mM Tris–HCl, pH7.5 for incubation; 500 mM NaCl, 0.5% NP-40, 50 mM Tris–HCl, pH7.5 for washing. Dithiothreitol (1 mM), RNase inhibitor (Superase-In, Life Technologies) and protease inhibitors (cOmplete, Roche Diagnostics, Indianapolis, IN, USA) were added to the buffer just prior to use.

### RIP-seq read mapping and quantification

For the *Drosophila* RIP-seq experiments, sequencing reads were filtered using ELAND and those that passed the quality standard (Chastity >0.6) were mapped using Bowtie to the genome plus annotated transcriptome of *D. melanogaster*[90]. Next, we used ERANGE software to count the reads that fall into existing gene models and to pile putative new exons [38]. Clusters of reads that were close to known genes were either assigned as new exons of known genes or identified as novel transcripts on the basis of the read mapping pattern. Furthermore, because a number of *Drosophila* snRNA genes have multiple (two to seven) paralogs in the genome, we allowed up to ten mapped loci for each read. Subsequently, the repetitive reads were randomly assigned to mapped locations. The ERANGE final RPKM (reads per kilobase per million reads) data were converted to raw read numbers for each gene by using the calculated total number of reads for each sequenced library and the length of each gene. For each pair of control-IP experiments, we defined the read depth of a transcript *d* as the square root of the sum of the squares of number of reads in control and IP: d = sqrt(Ctrl × Ctrl + IP × IP). Raw read numbers for each gene between control and IP were normalized against the median of enrichment ratios for all expressed genes (with d > 10). The HeLa cell RIP-seq experiments were performed in duplicates (two controls and two IPs) with paired-end sequencing technology. We therefore used standard *t*-tests from the Tophat/Cufflinks pipeline to analyze the human RIP-seq data [91]. The q values and expression difference scores from Tophat/Cufflinks analysis were directly used. The sequencing data are accessible at Gene Expression Omnibus [92] with the accession number GSE35842.

### Assignment of reads to *Drosophila* snRNAs

To calculate the enrichment ratios of snRNAs as shown in Figure 3 and Table S5 in Additional file 1, the total numbers of reads mapped to all paralogs of each snRNA species were pooled from both random hexamer primed libraries and oligo(dT) primed libraries (BAM files), and reads with mismatches were discarded. The following strategy is employed to assign reads to distinct snRNA paralogs. For U1, U4 and U5 snRNAs, reads overlapping the variable regions were identified from mapped RIP-seq BAM files, and reads with mismatches were discarded. For U2 snRNA, reads overlapping the four variable regions were used to calculate the fraction each isoform takes, then the total number of U2 reads (without mismatches) was redistributed according to the calculated fractions. (Details available on request; ZL and AGM, manuscript in preparation.)

### *Drosophila* histone mRNA read mapping

Since the *Drosophila* replication-dependent histone genes are highly repetitive, we mapped all the RIP-seq reads to a single unit of the repeat, allowing no mismatches or indels. Then the read numbers were normalized against the median ratios obtained as mentioned above.

### *In situ* hybridizations

Full length LU snRNA and CG4692 mRNA and their antisense transcripts were produced using the T7 *in vitro* transcription system (MEGAscript T7 Kit, Life Technologies), and labeled with digoxigenin-UTP (DIG). The DIG-labeled probes were hybridized to S2 cells and detected using the tyramide signal amplification kit (Life Technologies) as previously described [21].

### Gaussian mixture modeling

Gaussian mixture modeling was performed on log-transformed enrichment ratios for all the RNAs with a read depth >10. The normalmixEM function from the R package mixtools was used for the modeling [93]. Specifically, we restrained the number of normal distributions to two, and the two distributions were homoscedastic. For example: y < − normalmixEM(x, lambda = 0.5, mu = c(0, 2), sigma = (0.5)). Model fitting for all the six *Drosophila* RIP-seq experiments on canonical Sm proteins converged. However, the Tralpt RIP-seq data did not. Since the canonical Sm RIP-seq yields around 200 enriched RNAs on average, we therefore arbitrarily used the top 200 RNAs from the Tralpt RIP-seq for pairwise comparisons.

### Cluster analysis of RIP-seq data

Enrichment ratios for every transcript in each of the seven RIP-seq experiments were log transformed. Then these enrichment ratios were clustered by experiment (but not genes) using Cluster 3.0 [94]. All available similarity metrics and clustering methods from the Cluster package were tried and all gave similar tree topology. After clustering, the data were visualized using Java Treeview [95]. The aspect ratio of the whole data matrix was scaled to fit the presentation.

### Fisher’s exact test of the significance of overlap

A total of 5,296 (denoted as *N*) RNAs with read depth >10 was used as the superset. For each pair of comparison, with *a* and *b* enriched RNAs (let *a* ≤ b), there are *n* overlapped RNAs. The Fisher’s exact test *P*-value was calculated using the following R function: sum(dhyper(*n*:*a*, *b*, *N*-*b*, *a*, log = FALSE)) [96].

### Phylogenetic analysis

To identify the homologs of the newly discovered ncRNAs, we first examined the same syntenic block in other insect species. In addition, the *D. melanogaster* ncRNA sequences (including the promoter region, for LU snRNA) were used to BLAST against genome and transcriptome databases for homologs [97]. Candidates were examined for the presence of signature sequence elements. The recovered sequences were aligned using ClustalW2 [98]. The phylogenetic tree of the homologs was constructed using drawtree-0.1.3 [99].

### Meta-gene analysis of read density around splice junctions

One transcript from each *Drosophila* or human Sm-associated intron-containing mRNA was randomly selected. Only internal exon-intron boundaries were used in this analysis. Reads were mapped using TopHat to increase the coverage around splice junctions. Reads mapped within a fifty nucleotide radius from the splice sites were counted from the following control and IP libraries (only random hexamer primed ones): Lu003-Lu004 (VFP-SmD3), Lu007-Lu008 (VFP-SmD3), Lu011-Lu012 (VFP-SmE), Lu015-Lu016 (VFP-SmB), Lu023-Lu024 (SmB), Lu025-Lu026 (SmD3pt), Lu045-Lu046-Lu047-Lu048 (human SmB). Scripts used for the analysis are available upon request.

### Meta-gene analysis of read density along the entire gene length

One transcript from each *Drosophila* Sm-associated intron-containing mRNA was randomly selected. We manually determined the poly(A) site for each transcript. Read density along the gene length was extracted from wiggle files of the following data. The oligodT primed IP libraries were Lu002, Lu006, Lu010 and Lu014, and the random hexamer primed were Lu004, Lu008, Lu012 and Lu016. For each library preparation method, the reads for all enriched RNAs in four libraries were added and the coordinate adjusted to the poly(A) site. Read density was adjusted so that the maximum equals to 1. Read density as far as 1 kb from the poly(A) site was displayed. Scripts used for the analysis are available upon request.

### Quantitative reverse-transcription PCR

Immunoprecipitated RNA was reverse transcribed with SuperScript III (Invitrogen) and digested with RNase H. Quantitative reverse-transcription PCR was performed using the SYBR Green master mix (Fermentas, Pittsburgh, PA, USA) on an ABI PRISM 7700 system (Applied Biosystems, Carlsbad CA, USA) according to the manufacturer’s instructions. At least three biological replicates were performed for each experiment. RT-PCR primers are listed in Table S8 in Additional file 1. To test the significance of IP versus control for each RNA, we used one-sided *t*-test, assuming heteroscedasticity.

### CG3776 construct and transfection

The CG3776 mRNA coding sequence (without the stop codon) was first cloned into pDONR221 and then transferred into pAW vectors using the Gateway system (Life Technologies). The three point mutations within the putative U1 binding site were introduced using Q5 Site-Directed Mutagenesis Kit (New England Biolabs, Ipswich, MA, USA). The construct expressed hybrid mRNA containing the CG3776 coding sequence and SV40/polyA 3′ UTR. The constructs were transfected into S2 cells using electroporation (Amaxa Lonza, Basel, Switzerland). See Table S8 in Additional file 1 for the mutagenesis primers and realtime PCR primers.

### Measurement of poly(A) tail length

Poly(A)-containing reads derived from a selected set of examples from the RIP-seq datasets were identified and summarized (Figure S11 in Additional file 1). PCR-based PAT assay was performed essentially as described [64]. Primers are listed in Table S8 in Additional file 1.

### Analysis of U1-70 K RIP-seq data

The U1-70 K (two replicates) and Empty (four replicates) IP read files were downloaded from the modENCODE website [70]. Reads were then mapped to the *Drosophila* genome and quantified using the TopHat/Cufflinks pipeline. For normalization of UCSC track files (wiggle, bedgraph, and so on) a given genome was divided into approximately 5,000 bins, and reads mapping to each bin were extracted from the track files. Only bins with significant read coverage were retained for subsequent analysis. The median of the ratios between the corresponding bins in two track files was used as the normalization factor.

### RNA secondary structure and base pairing prediction

The secondary structures of the newly identified non-coding RNAs were predicted using either UNAfold or the Viena RNA Package with default parameter settings [100,101]. Secondary structures of the predicted RNAs were drawn using VARNA [102]. Structure alignment of ncRNAs was performed using LocARNA (global standard alignment) [103]. Single stranded regions of the known snRNAs were used to screen for mRNA sequence complementarity with these regions using RNAhybrid [104]. The minimum free energy was then calculated using the Vienna RNA package [101].

## Abbreviations

bp: Base pair; GFP: Green fluorescent protein; hTR: Human telomerase RNA; IP: Immunoprecipitation; mRNP: Messenger ribonucleoprotein; ncRNA: Non-coding RNA; PAR-CLIP: Photoactivatable-ribonucleoside-enhanced crosslinking and immunoprecipitation; PCR: Polymerase chain reaction; qRT-PCR: Quantitative reverse transcriptase PCR; RIP: RNA-immunoprecipitation; RNP: Ribonucleoprotein; scaRNA: Small Cajal body-specific RNA; snoRNA: Small nucleolar RNA; snRNA: Small nuclear RNA; snRNP: Small nuclear ribonucleoprotein; TMG: Trimethyl-guanosine; UTR: Untranslated region; VFP: Venus fluorescent protein.

## Competing interests

The authors declare that they have no competing interests.

## Authors’ contributions

ZL carried out most of the experiments and bioinformatic analyses. XG helped with the bioinformatics. CAS helped with the experiments in Figure 9. ZL and AGM designed the experiments and wrote the paper. All authors have read and approved the manuscript for publication.

## Supplementary Material

Additional file 1 **Inventory of supplementary information. Table S1:** details about the RIP-seq and RIP-qRT-PCR experiments (related to Figure 1d). **Table S2:** RIP-seq library statistics (related to Figure 1d). **Table S3:** mappable and unmappable read statistics in random hexamer primed libraries. **Table S4:** comparison of oligo(dT) and random hexamer primed libraries. **Table S5:** enrichment ratios of *Drosophila* Sm-associated RNAs (related to Figure 3a). **Table S6:** assignment of unique reads to *Drosophila* snRNA paralogs. **Table S7:** enrichment ratios of human Sm-associated RNAs (related to Figure 3b). **Table S8:** list of primers and oligos. **Figure S1:** per base quality of the RIP-seq data. **Figure S2:** additional scatterplots and Gaussian mixture modeling plots (related to Figure 2a,b). **Figure S3:** comparisons among all RIP-seq experiments, excluding ncRNAs (related to Figure 2d). **Figure S4:** enrichment ratios of the consensus set of Sm-associated RNAs. **Figure S5:** sequence alignment of *D. melanogaster* U1, U2, U4 and U5 paralogs. **Figure S6:** characterization of scaRNA:Prp8. **Figure S7:** alignments of LU promoters, Sm sites and 3′ ends with other snRNAs. **Figure S8:** genome browser view, structure, phylogeny and alignment of SHAN scaRNAs. **Figure S9:** CG4692 mRNA localization along oocyte cortex. **Figure S10:** enrichment ratios of *Drosophila* and human replication-dependent histone mRNAs. **Figure S11:** analysis of the polyadenylation of Sm-associated mRNAs (related to Figure 8b,d). **Figure S12:** Sm-associated mRNAs are not TMG-capped. **Figure S13:** additional predicted snRNP-mRNA base pairings (related to Figure 9a). Click here for file
