# Supplementary material for: RIP-seq analysis of eukaryotic Sm proteins identifies three major categories of Sm-containing ribonucleoproteins
Source: Genome Biol. 2014 Jan 7;15(1):R7. doi: 10.1186/gb-2014-15-1-r7 (PMC4053861; doi:10.1186/gb-2014-15-1-r7)
Supplement: Additional file 1 — Inventory of supplementary information. Table S1: details about the RIP-seq and RIP-qRT-PCR experiments (related to Figure 1d). Table S2: RIP-seq library statistics (related to Figure 1d). Table S3: mappable and unmappable read statistics in random hexamer primed libraries. Table S4: comparison of oligo(dT) and random hexamer primed libraries. Table S5: enrichment ratios of Drosophila Sm-associated RNAs (related to Figure 3a). Table S6: assignment of unique reads to Drosophila snRNA paralogs. Table S7: enrichment ratios of human Sm-associated RNAs (related to Figure 3b). Table S8: list of primers and oligos. Figure S1: per base quality of the RIP-seq data. Figure S2: additional scatterplots and Gaussian mixture modeling plots (related to Figure 2a,b). Figure S3: comparisons among all RIP-seq experiments, excluding ncRNAs (related to Figure 2d). Figure S4: enrichment ratios of the consensus set of Sm-associated RNAs. Figure S5: sequence alignment of D. melanogaster U1, U2, U4 and U5 paralogs. Figure S6: characterization of scaRNA:Prp8. Figure S7: alignments of LU promoters, Sm sites and 3′ ends with other snRNAs. Figure S8: genome browser view, structure, phylogeny and alignment of SHAN scaRNAs. Figure S9: CG4692 mRNA localization along oocyte cortex. Figure S10: enrichment ratios of Drosophila and human replication-dependent histone mRNAs. Figure S11: analysis of the polyadenylation of Sm-associated mRNAs (related to Figure 8b,d). Figure S12: Sm-associated mRNAs are not TMG-capped. Figure S13: additional predicted snRNP-mRNA base pairings (related to Figure 9a). [file gb-2014-15-1-r7-S1.docx]

Supplementary Information

**RNA immunoprecipitation sequencing (RIP-seq) analysis of eukaryotic Sm proteins identifies three major categories of Sm-containing ribonucleoproteins**

**Zhipeng Lu, Xiaojun Guan, Casey A. Schmidt and A. Gregory Matera**

**Inventory of supplementary information**

Supplementary Table 1. Details about the RIP-seq and RIP-qRT-PCR experiments (related to Fig. 1d)

Supplementary Table 2. RIP-seq library statistics (related to Fig. 1d)

Supplementary Table 3. Mappable and unmappable read statistics in random hexamer primed libraries.

Supplementary Table 4. Comparison of oligo(dT) and random hexamer primed libraries.

Supplementary Table 5. Enrichment ratios of *Drosophila* Sm-associated RNAs (related to Fig. 3a)

Supplementary Table 6. Assignment of unique reads to *Drosophila* snRNA paralogs

Supplementary Table 7. Enrichment ratios of human Sm-associated RNAs (related to Fig. 3b)

Supplementary Table 8. List of primers and oligos

Supplementary Figure 1. Per base quality of the RIP-seq data

Supplementary Figure 2. Additional Scatterplots and Gaussian mixture modeling plots (related to Fig. 2a,b)

Supplementary Figure 3. Comparisons among all RIP-seq experiments, excluding ncRNAs (related to Fig. 2d)

Supplementary Figure 4. Enrichment ratios of the consensus set of Sm-associated RNAs.

Supplementary Figure 5. Sequence alignment of *D. melanogaster* U1, U2, U4 and U5 paralogs.

Supplementary Figure 6. Characterization of scaRNA:Prp8.

Supplementary Figure 7. Alignments of LU promoters, Sm sites and 3’ ends with other snRNAs.

Supplementary Figure 8. Genome browser view, structure, phylogeny and alignment of SHAN scaRNAs.

Supplementary Figure 9. CG4692 mRNA localization along oocyte cortex.

Supplementary Figure 10. Enrichment ratios of *Drosophila* and human replication-dependent Histone mRNAs.

Supplementary Figure 11. Analysis of the polyadenylation of Sm-associated mRNAs (related to Fig. 7b,c and d).

Supplementary Figure 12. Sm-associated mRNAs are not TMG-capped.

Supplementary Figure 13. Additional predicted snRNP-mRNA base pairings (related to Fig. 9a)

**Supplementary Tables**

| **Fly strain** | **Antibody** | **Expression** | **Priming** | | **Sample No.** |
| --- | --- | --- | --- | --- | --- |
| *Nos-Gal4 VFP-SmD3* | αGFP | germline | Exp1 | Oligo dT | Lu001(Ctrl), Lu002(IP) |
|  |  |  |  | 6mer | Lu003(Ctrl), Lu004(IP) |
|  |  |  | Exp2 | Oligo dT | Lu005(Ctrl), Lu006(IP) |
|  |  |  |  | 6mer | Lu007(Ctrl), Lu008(IP) |
| *SmD3pt* | αGFP | ubiquitous | 6mer | | Lu025(Ctrl), Lu026(IP) |
| *Nos-Gal4 VFP-SmB* | αGFP | germline | Oligo dT | | Lu013(Ctrl), Lu014(IP) |
|  |  |  | 6mer | | Lu015(Ctrl), Lu016(IP) |
| *Oregon R* | Y12 | ubiquitous | 6mer | | Lu023(Ctrl), Lu024(IP) |
| *Nos-Gal4 VFP-SmE* | αGFP | germline | Oligo dT | | Lu009(Ctrl), Lu010(IP) |
|  |  |  | 6mer | | Lu011(Ctrl), Lu012(IP) |
| *Da-Gal4 VFP-SmD1* | αGFP | ubiquitous | 6mer | | qRT-PCR |
| S2 cells | Y12 | – | 6mer | | qRT-PCR |
| HeLa cells | Y12 | – | 6mer | | Lu045 and Lu046 (Ctrl)  Lu047 and Lu048 (Ctrl) |

**Supplementary Table 1.** Details about the RIP-seq and RIP-qRT-PCR experiments (related to Fig. 1d)

6mer: random hexamer. The RIP-seq experiments on nos-Gal4 VFP-SmD3 fly ovaries were performed as biological replicates. All of these RIP-seq experiments were performed on different days.

| **samples** | **sample No.** | **unique** | **spliced** | **RNAFAR** | **multi** | **mapped** | **total reads** | **mappability** | **adjust** |
| --- | --- | --- | --- | --- | --- | --- | --- | --- | --- |
| nos Gal4  VFP-SmD3 single-end | Lu001 | 39148 | 1118 | 144 | 107024 | 147434 | 10426289 | 0.014140602 |  |
|  | Lu002 | 106073 | 2199 | 869 | 5070549 | 5179690 | 8063387 | 0.6423715 | 1.57 |
|  | Lu003 | 316701 | 14664 | 1434 | 100558 | 433357 | 11666984 | 0.037143875 |  |
|  | Lu004 | 1314704 | 61607 | 12153 | 11152871 | 12541335 | 18950426 | 0.661796996 | 3.46 |
| nos Gal4  VFP-SmD3 single-end | Lu005 | 12986 | 384 | 131 | 202801 | 216302 | 19219893 | 0.011254069 |  |
|  | Lu006 | 59348 | 1479 | 531 | 3145331 | 3206689 | 20415816 | 0.157068863 | 3.32 |
|  | Lu007 | 137627 | 6658 | 603 | 171802 | 316690 | 19641080 | 0.016123859 |  |
|  | Lu008 | 469701 | 20478 | 3860 | 3755700 | 4249739 | 23267823 | 0.182644461 | 2.28 |
| nos Gal4  VFP-SmE single-end | Lu009 | 339674 | 16882 | 1420 | 33122 | 391098 | 13775633 | 0.028390565 |  |
|  | Lu010 | 1675331 | 84927 | 11474 | 111670 | 1883402 | 21298219 | 0.088430023 | 5.62 |
|  | Lu011 | 555161 | 28406 | 1296 | 39507 | 624370 | 20733108 | 0.030114636 |  |
|  | Lu012 | 1343009 | 70417 | 8133 | 76377 | 1497936 | 22561102 | 0.066394629 | 2.46 |
| nos Gal4  VFP-SmB single-end | Lu013 | 195195 | 5139 | 1620 | 55055 | 257009 | 23034642 | 0.011157499 |  |
|  | Lu014 | 2415750 | 87425 | 33291 | 130114 | 2666580 | 23043259 | 0.115720611 | 10.84 |
|  | Lu015 | 418808 | 18375 | 903 | 40119 | 478205 | 19020871 | 0.025141067 |  |
|  | Lu016 | 3866621 | 186827 | 29650 | 155750 | 4238848 | 21121289 | 0.200690782 | 8.63 |
| Tralpt  single-end | Lu019 | 4519439 | 241175 | 26155 | 355607 | 5142376 | 29032697 | 0.177123607 |  |
|  | Lu020 | 8798978 | 444498 | 89240 | 392601 | 9725317 | 28358846 | 0.342937685 |  |
| Oregon R single-end | Lu023 | 799995 | 39024 | 6860 | 203022 | 1048901 | 23128732 | 0.045350562 |  |
|  | Lu024 | 3905235 | 219000 | 37621 | 1131728 | 5293584 | 27762576 | 0.190673373 | 3.95 |
| SmD3pt single-end | Lu025 | 3273832 | 167489 | 89240 | 205543 | 3736104 | 28031693 | 0.133281425 |  |
|  | Lu026 | 8265251 | 430878 | 105031 | 1226538 | 10027698 | 24649805 | 0.406806382 | 2.13 |
| HeLa cells paired-end | Lu045 | – | – | – | 28212679 | 30598572 | 32324208 | 0.946614748 |  |
|  | Lu046 | – | – | – | 25909287 | 27795460 | 28647941 | 0.970242853 | 0.8402 |
|  | Lu047 | – | – | – | 37043623 | 38550693 | 39243007 | 0.982358284 | 0.5779 |
|  | Lu048 | – | – | – | 38302969 | 39946114 | 41314245 | 0.966884763 | 0.6545 |

**Supplementary Table 2.** RIP-seq library statistics (related to Fig. 1d). RNAFAR: Reads that cluster to putative new genes or new exons of known genes. Adjust: the adjustment (normalization) factors between each pair of Ctrl and IP (i.e. raw read numbers from the IP in each pair should be divided by the adjustment factor to give normalized read numbers).

| **library** | **Note** | **Mappable (%)** | **Primers (%)** | **rRNA (%)** | **Other (%)** | **Total (%)** |
| --- | --- | --- | --- | --- | --- | --- |
| VFP-SmD3_Lu003 | Non-size-selected | 3.7 | 78.1 | 6.4 | 11.8 | 100 |
| VFP-SmD3_Lu004 |  | 66.2 | 11.2 | 11.5 | 11.1 | 100 |
| VFP-SmD3_Lu007 |  | 1.6 | 81.9 | 1.5 | 15.0 | 100 |
| VFP-SmD3_Lu008 |  | 18.3 | 56.2 | 1.1 | 24.4 | 100 |
| VFP-SmE_Lu011 | Size-selected | 3.0 | 0.0 | 48.3 | 48.7 | 100 |
| VFP-SmE_Lu012 |  | 6.6 | 0.0 | 53.1 | 40.3 | 100 |
| VFP-SmB_Lu015 |  | 2.5 | 0.0 | 37.1 | 60.4 | 100 |
| VFP-SmB_Lu016 |  | 20.1 | 0.0 | 36.6 | 43.3 | 100 |
| Tralpt_Lu019 |  | 17.7 | 1.4 | 24.1 | 56.8 | 100 |
| Tralpt_Lu020 |  | 34.3 | 1.6 | 7.2 | 56.9 | 100 |
| SmB_Lu023 |  | 4.5 | 2.5 | 22.0 | 71.0 | 100 |
| SmB_Lu024 |  | 19.1 | 1.7 | 14.5 | 64.7 | 100 |
| SmD3pt_Lu025 |  | 13.3 | 1.4 | 30.7 | 54.6 | 100 |
| SmD3pt_Lu026 |  | 40.7 | 2.3 | 18.6 | 38.5 | 100 |

**Supplementary Table 3.** Mappable and unmappable read statistics in random hexamer primed libraries.

The random hexamer-primed libraries were used for the enrichment analysis presented in the main text. The first four libraries were prepared without size-selection, whereas the latter ones were size-selected to remove primer-dimers. The rRNA reads were quantified by mapping total reads to a single copy of the 45S rRNA repeat unit and 5S rRNA repeat unit, allowing no-mismatches. The reads derived from primes/adaptors were quantified using the fast-qc program. Taken together, these three categories encompass 30% - 90% of each library, whereas the balance (i.e. the “Other” column) are unmappable reads. The vast majority of reads in the ‘Other’ category are random sequences that do not match to any known genome, along with a few trace contaminants (e.g. bacterial *Propionibacterium* *acnes*, or rainbow trout, *Oncorhynchus* *myki*s*s* sequences). Among each pair of experiments shown in the table, the rRNA and primer-dimer reads are more abundant in the control libraries (odd numbers) than they are in the IP libraries (even numbers). This is to be expected due to the limited amount of RNA brought down in the IPs (or control/mock IPs).

| **Experiments** | **No. RNAs (d>10)** | | **mRNA reads** | | **snRNA reads** | |
| --- | --- | --- | --- | --- | --- | --- |
|  | **OligodT** | **6mer** | **OligodT** | **6mer** | **OligodT** | **6mer** |
| Lu001-Lu004 | 1478 | 6545 | 57603 | 706143 | 3250538 | 3352434 |
| Lu005-Lu008 | 693 | 5384 | 13111 | 301516 | 1151580 | 1840716 |
| Lu009-Lu012 | 7065 | 6780 | 653812 | 1175978 | 11002 | 24683 |
| Lu013-Lu016 | 6980 | 7087 | 394807 | 919921 | 13805 | 13499 |

**Supplementary Table 4.** Comparison of oligo(dT) and random hexamer primed libraries. Oligo(dT) primed libraries produced fewer mRNAs with significant coverage, and fewer reads for mRNAs.

| **Gene ID** | | **Annotation** | **Enrichment ratios** | | | | | |
| --- | --- | --- | --- | --- | --- | --- | --- | --- |
|  |  |  | **VFP-SmD3** | **VFP-SmD3** | **VFP-SmE** | **VFP-SmB** | **SmB** | **SmD3pt** |
| **snRNAs** | |  |  |  |  |  |  |  |
| Five U1 | | U1 | 28.9 | 7.8 | 2.4 | 110.2 | 84.2 | 633.5 |
| Six U2 | | U2 | 59.0 | 111.3 | 87.1 | 46.0 | 108.7 | 459.1 |
| Three U4 | | U4 | 109.3 | 120.3 |  | 3.4 | 107.8 | 191.6 |
| Seven U5 | | U5 | 78.7 | 93.6 | 8.8 | 7.1 | 124.6 | 81.4 |
| Three U6 | | U6 | 97.8 | 59.6 | 13.0 | 5.1 | 96.9 | 69.1 |
| CR34151 | | U11 | 167.0 | 290.7 | 96.9 | 70.1 | 484.1 | 433.9 |
| CR32162 | | U12 | 72.4 | 299.3 | 92.4 | 137.8 | 155.1 | 329.3 |
| CR32860 | | U4atac | 31.4 | 66.4 |  |  | 79.0 | 103.6 |
| CR32989 | | U6atac | 6.3 | 6.0 |  |  | 6.6 | 5.7 |
| CR43708 | | LU | 135.7 | 530.6 | 3.6 | 5.1 | 29.8 | 27.9 |
| **scaRNAs** | |  |  |  |  |  |  |  |
| CR32863 | | snoRNA:MeU5-C46 (U85) | 10.1 | 1.9 | 1.7 | 14.1 | 4.3 | 9.4 |
| CR33716 | | snoRNA:MeU5-U42 | 4.6 | 3.1 |  |  |  |  |
| CR43600 (new) | | scaRNA:Prp8 | 10.1 | 4.1 |  |  |  | 8 |
| **Mitochondrial** | |  |  |  |  |  |  |  |
| CG4692 | | ATP synthase | 32.5 | 37.2 | 7.5 | 7.3 | 15.9 | 28 |
| CG3776 | | Jhebp29 | 8.9 | 6.8 | 3.2 | 46.5 | 18.1 | 63.9 |
| CG13410 | | mRpL35 | 12.1 | 17 | 3.5 | 41.9 | 11.5 | 17.7 |
| CG13240 | | NADH dehydrogenase subunit | 11.6 | 20.3 | 6.3 | 6.8 | 20.8 | 23.6 |
| CG1349 | | dj-1beta/PARK7 | 9.9 | 14.1 | 7.4 | 6.1 | 8.4 | 17.5 |
| CG8043 | | IBA57, Fe/S assembly | 3.2 | 18.3 | 3.1 | 34.2 | 10.5 | 9.7 |
| CG14806 | | Apopt1 | 6.7 | 3.7 | 5.5 | 11.4 | 10.8 | 7.7 |
| CG9065 | | cox17 | 2.8 | 4.1 | 4.3 | 21.6 | 15.2 | 7.5 |
| CG11968 | | Ras-related GTP binding A | 8.4 | 4.7 | 3.8 | 8.4 | 8.6 | 7.6 |
| CG13393 | | DAD1, phospholipase | 4.2 | 5.5 | 3.3 | 9.8 | 7.8 | 9.4 |
| CG2098 | | ferrochelatase | 8.8 | 4.4 | 3 | 6.1 | 9.9 | 7.8 |
| CG18624 | | NADH dehydrogenase subunit | 3.3 | 4.6 | 2.1 | 21 | 11 | 7.2 |
| CG9291 | | elongin-C | 6.7 | 8.4 | 4.6 | 2.3 | 11.4 | 7.4 |
| CG10009 | | NOA36/ZNF330 | 4.1 | 4.5 | 4 | 6.9 | 10.1 | 9.3 |
| CG6008 | | NADH dehydrogenase subunit | 4.5 | 4.5 | 4.5 | 7.4 | 5.1 | 13.5 |
| CG31450 | | mRpS18A | 4.3 | 9.5 | 4.1 | 2.6 | 8.6 | 9.3 |
| CG33714 | | RNA binding protein | 5 | 7.9 | 2.6 | 4.1 | 7.8 | 6.9 |
| CG3552 | | GDP-D-glucose phosphorylase | 4.5 | 4.5 | 2.2 | 15.3 | 4.7 | 2.6 |
| CG9160 | | mtacp1 | 4.8 | 4.7 | 3 | 5.1 | 4.5 | 5 |
| CG2915 | | carboxypeptidase M14 like | 3.6 | 5 | 2.2 | 4.2 | 5.8 | 4 |
| **Translation** |  | |  |  |  |  |  |  |
| CG3997 | RpL39 | | 6.8 | 17 | 1.4 | 70.7 | 24.7 | 8.9 |
| CG8857 | RpS11 | | 8 | 10.4 | 1.8 | 16 | 14.8 | 7.8 |
| CG6141 | RpL9 | | 5.6 | 6.2 | 1.9 | 14.8 | 15.7 | 9.5 |
| CG17420 | RpL15 | | 4.5 | 7.1 | 2.4 | 6.2 | 24.3 | 9.9 |
| CG1475 | RpL13A | | 4.8 | 9.9 | 2 | 14 | 8 | 9 |
| CG7993 | rpf2, ribosome production | | 4.5 | 7.6 | 2.3 | 8.5 | 8.2 | 10.8 |
| CG5032 | rRNA methyltransferase | | 5.7 | 4.1 | 2 | 12.1 | 9.2 | 7.5 |
| CG6937 | MKI67IP, ribosome biogenesis | | 4.6 | 6.4 | 3.4 | 5.5 | 9.4 | 5.5 |
| CG7137 | rRNA-processing protein 8 | | 5.8 | 7.1 | 2.4 | 7.7 | 4.7 | 3.4 |
| CG7283 | RpL10Ab | | 4.2 | 5.2 | 1.2 | 8.9 | 6.1 | 7.5 |
| CG5271 | RpS27A | | 5.1 | 6.7 | 1.3 | 5.4 | 5.2 | 3.5 |
| CG7883 | eIF2Balpha | | 8 | 6.1 | 4.6 | 14.2 | 13.5 | 11.2 |
| CG8005 | Dhps, eIF5A modification | | 4 | 2.3 | 5.2 | 6.5 | 4.9 | 5.4 |

Supplementary Table 5. Enrichment ratios of *Drosophila* Sm-associated RNAs (related to Table 3a). U1, U2, U4, U5 and U6 are multi-copy snRNAs, and for each snRNA species, all reads mapped to all the paralogs are pooled for calculation of enrichment ratios. Please refer to the methods section for details of the analysis. Please refer to Supplementary Table 5 for assignment of reads to distinguishable snRNA paralogs. Empty spaces means that there are not enough reads for that particular snRNA or scaRNA. Enrichment of the scaRNAs and mRNAs as determined by Gaussian mixture modeling is indicated by the highlighting. Note: even though these RNAs are not deemed significantly enriched in some of the samples (unhighlighted cells), their enrichment ratios are mostly larger than 1.

| **Gene ID** | **Annotation** | **Enrichment ratios** | | | | | |
| --- | --- | --- | --- | --- | --- | --- | --- |
|  |  | **VFP-SmD3** | **VFP-SmD3** | **VFP-SmE** | **VFP-SmB** | **SmB** | **SmD3pt** |
| **Miscellaneous** |  |  |  |  |  |  |  |
| CG6153 | pithd1 | 12.4 | 10.8 | 7.3 | 17.1 | 12.7 | 10.7 |
| CG13951 | Zfp511 | 7 | 7.6 | 3.4 | 52.6 | 17.9 | 7.6 |
| CG12173 | enoph1 | 5.5 | 6.7 | 9.9 | 14.2 | 13.3 | 14.9 |
| CG18278 | N-acetylglucosamine-6-sulfatase | 5.8 | 5.4 | 5.6 | 39.7 | 18.2 | 4.1 |
| CG30059 | N-acetylglucosamine-6-sulfatase | 25.3 | 17.4 | 1.8 | 30.4 | 5.1 | 3.6 |
| CG4789 | Rabl3 | 3.8 | 8.7 | 4.1 | 25.2 | 9 | 14.3 |
| CG2261 | CstF-50, WD40 | 10.5 | 8.6 | 3.2 | 10.2 | 11.7 | 12.7 |
| CG5325 | Pex19 | 14.5 | 7.3 | 3.9 | 6.3 | 12.5 | 12.4 |
| CG4645 | yipf1 | 9 | 6.4 | 2.3 | 7 | 19.9 | 15.9 |
| CG9953 | peptidase s28 | 7.1 | 5.8 | 2.9 | 15.8 | 12.1 | 11.2 |
| CG14341 | only in arthropods | 3.7 | 20.1 | 2.9 | 19.5 | 6.2 | 8.4 |
| CG17531 | GstE | 9.2 | 4.8 | 2.2 | 13.8 | 6.9 | 11.8 |
| CG10053 | ccdc75/CENP-Y | 8.1 | 2.7 | 3.6 | 10.2 | 13.2 | 9.5 |
| CG5972 | arp-p20 | 11 | 7 | 4.4 | 4.3 | 5.2 | 11.7 |
| CG17294 | HDHD2 | 2.6 | 5.4 | 1.3 | 13.6 | 17.3 | 19.5 |
| CG6363 | MRG15 | 5.6 | 6.3 | 2.8 | 8 | 12.9 | 7 |
| CG4775 | Tango14, alkyl/aryl transferase | 3.1 | 5.6 | 2.3 | 38.2 | 5.9 | 7.6 |
| CG9526 | frj, mboat family protein | 5.4 | 6.3 | 2.6 | 12.2 | 7.6 | 8.2 |
| CG15309 | Yippee-like | 9.7 | 10.2 | 1.2 | 2 | 22.8 | 11 |
| CG14187 | only in Drosophila species | 4.7 | 6.4 | 1.3 | 7.7 | 14.6 | 11.5 |
| CG8727 | Cycle/dBMAL | 5.2 | 6 | 2.6 | 9.2 | 8.2 | 6.9 |
| CG30105 | rnaseH2 | 7.2 | 4.3 | 3.1 | 3.3 | 10.6 | 10.8 |
| CG18764 | only in Drosophila species | 5.3 | 3.4 | 4.9 | 21.2 | 4.9 | 3.5 |
| CG10728 | vls | 6.4 | 6.3 | 3.2 | 3.7 | 7.2 | 8 |
| CG2790 | Hsp70 binding | 5.9 | 4 | 3.6 | 6.9 | 9.3 | 4.7 |
| CG2611 | only in arthropods, DUF872 | 3.8 | 3.9 | 4 | 9.8 | 6.3 | 6.2 |
| CG12357 | CBP20 | 4.2 | 4.1 | 3.1 | 8.6 | 13 | 3.3 |
| CG17765 | Ca-binding, EF hand | 6 | 3.9 | 3.1 | 4.8 | 8.1 | 6.9 |
| CG33713 | Acbd6, acyl-CoA binding | 4.8 | 7 | 2.6 | 4.1 | 7.8 | 6.9 |
| CG7405 | cyclin H | 5.6 | 7.2 | 3.3 | 3.7 | 6.1 | 6.4 |
| CG17322 | UDP glucosyl transferase | 5.2 | 2.2 | 3.3 | 9.9 | 6.1 | 5.7 |
| CG11076 | only in Drosophila species | 4 | 5.8 | 2.2 | 6.7 | 8.6 | 3.9 |
| CG5808 | PPIL4, cyclophilin | 3.7 | 2.8 | 3.8 | 6.7 | 7.3 | 5.2 |
| CG9742 | SmG | 5.5 | 7.8 | 3.1 | 5.3 | 4.4 | 3 |
| CG8735 | LNP1, zinc finger, transmembrane | 3.6 | 6.4 | 1.4 | 8 | 7.3 | 4.8 |
| CG13151 | AT-hook, DNA binding | 4.5 | 4.2 | 5.1 | 1.4 | 8 | 7.6 |
| CG17347 | dynactin6 | 6.3 | 5.9 | 2.9 | 5.4 | 9.6 | 1 |
| CG9752 | c9orf64 | 5 | 5.5 | 0.8 | 1.9 | 10.3 | 7.3 |
| CG13737 | only in Drosophila species | 3.2 | 4.6 | 1.5 | 5.6 | 2.4 | 9 |

Supplementary Table 5 continued. Enrichment ratios of *Drosophila* Sm-associated RNAs (related to Table 3a)

|  | **VFP-SmD3_1**  **Oligo(dT)** | | **VFP-SmD3_1**  **6mer** | | **VFP-SmD3_2**  **Oligo(dT)** | | **VFP-SmD3_2**  **6mer** | | **VFP-SmE**  **Oligo(dT)** | | **VFP-SmE**  **6mer** | | **VFP-SmB**  **Oligo(dT)** | | **VFP-SmB**  **6mer** | | **SmB**  **6mer** | | **SmD3pt**  **6mer** | |  |
| --- | --- | --- | --- | --- | --- | --- | --- | --- | --- | --- | --- | --- | --- | --- | --- | --- | --- | --- | --- | --- | --- |
| **snRNA paralogs** | **Lu001** | **Lu002** | **Lu003** | **Lu004** | **Lu005** | **Lu006** | **Lu007** | **Lu008** | **Lu009** | **Lu010** | **Lu011** | **Lu012** | **Lu013** | **Lu014** | **Lu015** | **Lu016** | **Lu023** | **Lu024** | **Lu025** | **Lu026** | |
| U1:21D,U1:95Ca,U1:95Cb | 2676 | 71161 | 1785 | 193643 | 4824 | 27630 | 4467 | 39845 | 15 | 49 | 9 | 26 | 0 | 42 | 0 | 26 | 1692 | 85180 | 330 | 93882 | |
| U1:82Eb | 278 | 16868 | 330 | 31305 | 1069 | 7143 | 880 | 9741 | 1 | 6 | 5 | 1 | 0 | 6 | 0 | 3 | 58 | 3068 | 21 | 4932 | |
| U1:95Cc | 44 | 1852 | 37 | 3923 | 117 | 868 | 92 | 1275 | 0 | 2 | 0 | 2 | 0 | 1 | 0 | 0 | 22 | 1329 | 8 | 1869 | |
| U2:14B | 531 | 26255 | 654 | 43704 | 84 | 7087 | 69 | 10009 | 1 | 380 | 6 | 292 | 9 | 188 | 0 | 214 | 110 | 11935 | 94 | 43015 | |
| U2:34ABa | 1766 | 87235 | 2172 | 145213 | 281 | 23548 | 229 | 33255 | 4 | 1262 | 21 | 970 | 29 | 623 | 0 | 713 | 364 | 39656 | 311 | 142924 | |
| U2:34ABb,U2:34ABc | 5017 | 247854 | 6171 | 412580 | 797 | 66904 | 651 | 94485 | 11 | 3586 | 60 | 2755 | 81 | 1771 | 0 | 2025 | 1035 | 112672 | 883 | 406078 | |
| U2:38ABa | 333 | 16440 | 409 | 27367 | 53 | 4438 | 43 | 6267 | 1 | 238 | 4 | 183 | 5 | 117 | 0 | 134 | 69 | 7474 | 59 | 26936 | |
| U2:38ABb | 67 | 3309 | 82 | 5508 | 11 | 893 | 9 | 1261 | 0 | 48 | 1 | 37 | 1 | 24 | 0 | 27 | 14 | 1504 | 12 | 5421 | |
| U4:25F | 0 | 13 | 0 | 12 | 0 | 5 | 0 | 9 | 0 | 0 | 0 | 0 | 0 | 0 | 0 | 0 | 0 | 7 | 0 | 19 | |
| U4:38AB | 1 | 156 | 0 | 295 | 0 | 63 | 0 | 134 | 0 | 0 | 0 | 0 | 0 | 0 | 0 | 1 | 0 | 104 | 0 | 241 | |
| U4:39B | 5 | 268 | 3 | 386 | 1 | 70 | 1 | 146 | 0 | 1 | 0 | 1 | 0 | 1 | 0 | 2 | 0 | 174 | 1 | 250 | |
| U5:14B | 0 | 43 | 0 | 56 | 0 | 3 | 0 | 11 | 0 | 0 | 0 | 0 | 0 | 0 | 0 | 0 | 0 | 4 | 0 | 0 | |
| U5:23D | 0 | 73 | 1 | 69 | 0 | 5 | 0 | 18 | 0 | 0 | 0 | 0 | 0 | 0 | 0 | 0 | 0 | 5 | 0 | 3 | |
| U5:34A | 0 | 70 | 0 | 13 | 0 | 2 | 0 | 4 | 0 | 0 | 0 | 0 | 0 | 0 | 0 | 0 | 0 | 5 | 0 | 0 | |
| U5:35D | 0 | 6 | 0 | 3 | 0 | 1 | 0 | 0 | 0 | 0 | 0 | 0 | 0 | 0 | 0 | 0 | 0 | 0 | 0 | 0 | |
| U5:38ABa | 0 | 49 | 2 | 47 | 0 | 4 | 0 | 7 | 0 | 0 | 0 | 0 | 0 | 0 | 0 | 0 | 0 | 1 | 0 | 1 | |
| U5:38ABb | 0 | 55 | 0 | 2 | 0 | 0 | 0 | 0 | 0 | 0 | 0 | 0 | 0 | 0 | 0 | 0 | 0 | 1 | 0 | 0 | |
| U5:63BC | 1 | 195 | 6 | 165 | 2 | 12 | 1 | 37 | 0 | 0 | 0 | 0 | 0 | 0 | 0 | 0 | 0 | 2 | 0 | 2 | |

Supplementary Table 6. Assignment of unique reads to *Drosophila* snRNA paralogs (except U2, which is reassignment of all reads, please see the methods section for details). snRNA paralogs with identical sequences are listed in one row. The highlighted columns are the IPs, whereas the unhighlighted ones are the controls.

| **gene** | **Annotation** | **Enrichment ratio** | **q_value** |
| --- | --- | --- | --- |
| **snRNAs** |  |  |  |
| U1 |  | 237 |  |
| U2 |  | 90 |  |
| U4 |  | 305 |  |
| U5 |  | 330 |  |
| U6 |  | 26 |  |
| U4atac |  | 550 |  |
| U6atac |  | 373 |  |
| U11 |  | 343 |  |
| U12 |  | 325 |  |
| **scaRNAs** |  |  |  |
| SCARNA2 | C/D box, HBII-382, mgU2-25/61 | 2.1 | 5.2E-02 |
| SCARNA5 | C/D box, U87, mgU4-A65/mgU5-U41 | 5.1 | 1.2E-01 |
| SCARNA9 | C/D box, Z32, mgU2-G19/A30 | 9.7 | 6.7E-02 |
| SCARNA10 | C/D and H/ACA box, U85, psiU5-U46/mgU5-C45 | 2.6 | 8.3E-02 |
| SCARNA12 | H/ACA box, U89, psiU5-U46 | 2.8 | 1.7E-02 |
| SCARNA16 | H/ACA box, ACA47, psiU1-U5 | 15.4 | 0 |
| SCARNA17 | C/D box, mgU12-22/U4-8 | 7.1 | 4.8E-02 |
| SNORD118 | C/D box, U8, | 3.9 | 6.8E-02 |
| SHAN (new) | H/ACA box, tRNA_Asp, target unknown | 38.2 | 6.7E-02 |
| **mRNAs** |  |  |  |
| HIST2H2AB | Replication-dependent histone | 9.7 | 1.1E-07 |
| HIST1H2AM | Replication-dependent histone | 2.6 | 1.1E-02 |
| RPL23 | Ribosomal protein | 5.6 | 0 |
| RPS6 | Ribosomal protein | 2.1 | 1.2E-03 |
| RP1-278E11.3 | Ribosomal protein pseudogene | 5.0 | 4.7E-05 |
| EIF3G | translation | 4.1 | 4.4E-07 |
| MCAT | Malonyl CoA-acyl carrier protein transacylase | 6.9 | 1.7E-03 |
| NQO2 | NAD(P)H dehydrogenase, quinone 2 | 3.2 | 4.7E-04 |
| PFKM | muscle phosphofructokinase | 3.6 | 1.0E-06 |
| UQCRC2 | ubiquinol-cytochrome c reductase core protein II | 2.3 | 1.1E-03 |
| LDOC1L | leucine zipper, down-regulated in cancer 1-like | 6.0 | 1.4E-04 |
| TAF5L | Pol II transcription factor associated protein | 6.0 | 1.0E-02 |
| FLYWCH2 | zinc finger protein | 5.4 | 4.4E-08 |
| FKBP2 | ER chaperone | 9.7 | 6.1E-10 |
| PKD1P1 | pseudogene | 5.3 | 2.1E-02 |
| c16orf5 | cell death inducing protein, | 4.6 | 7.8E-05 |
| KLHL12 | Ubiquitination | 3.7 | 2.0E-02 |
| TBCB | Tubulin-folding cofactor B | 3.6 | 4.8E-04 |
| CDCA7L | cell division cycle-associated 7-like protein | 3.2 | 1.2E-03 |
| FSTL3 | Follistatin-related protein 3 | 2.9 | 8.8E-04 |
| DNER | Delta and Notch-like EGF-related receptor | 2.7 | 2.9E-03 |
| CCT5 | chaperonin containing TCP1, subunit 5 (epsilon) | 2.6 | 9.1E-05 |
| AVPI1 | Arginine vasopressin-induced protein 1 | 2.5 | 2.2E-04 |
| WDR1 | WD40 repeat protein | 2.5 | 1.4E-04 |
| ERGIC3 | ER-Golgi intermediate compartment protein 3 | 2.3 | 9.9E-04 |
| ASNS | asparagine synthase | 2.3 | 4.4E-04 |
| ADSL | adenylosuccinate lyase | 2.3 | 4.9E-02 |
| ATG13 | autophagy gene 13 | 2.3 | 3.0E-02 |
| SPTBN2 | spectrin, beta, non-erythrocytic 2 | 2.2 | 4.3E-02 |
| CTSL1 | Cathepsin L1,  lysosomal cysteine proteinase | 2.2 | 1.1E-02 |
| LAMA5 | laminin alpha5, extracellular matrix | 2.2 | 1.4E-02 |
| TARDBP | FTLD, ALS | 2.1 | 7.9E-03 |
| AGRN | agrin | 2.1 | 3.4E-02 |
| GSTP1 | related to Drosophila GST | 2.0 | 1.2E-02 |
| SH3BP4 | clathrin-mediated endocytosis | 1.9 | 2.0E-02 |

**Supplementary Table 7.** Enrichment ratios of human Sm-associated RNAs (related to Table 3b). Note the enrichment ratios for the snRNAs are from the medians of mappable human snRNAs.

| Gene | Forward (5’-3’) | Reverse (5’-3’) | Size (bp) |
| --- | --- | --- | --- |
| U1:21D | ATACTTACCTGGCGTAGAGGTTAACC | GGAATGGCGTTCGCGCCGTCCCGA | 164 |
| U2:14B | ATCGCTTCTCGGCCTTATGGCTAAGATC | GTTGGGCCGAAGTCCCGGCGGTACTGCA | 192 |
| LU | ATGTCTCGATCGCCGCTTCAGTTGT | AATTGCCTCGGATAATGTGCTCATC | 80 |
| scaRNA:Prp8 | ACGTTTCCAAGTGATCAGCCTCTCTGG | ATATGTATGCAACTATCAGCAGTCACGAT | 168 |
| 5S rRNA | GCCAACGACCATACCACGCTGAA | AGTTGTGGACGAGGCCAACAACAC | 120 |
| Act5C | CGTCTTCCCATCGATTGTGGGACGT | AGTCGGTCAAATCGCGACCAGCCAGA | 476 |
| Smt3 | CGGCATTCGACGCTCCGCAA | ATGGAGCGCCACCAGTCTGC | 350 |
| CG1349 | TGTCGAAAAGCGCGCTGGTGAT | CGGCTACGGTGACCTTGATGCC | 105 |
| CG3776 | CGGGAACGCGGCGAGGAAAT | CCGATTGGTGTCCAGCGGTGA | 115 |
| CG3776 mutagenesis | cggaatTATGTTTGACGATGCAAAC | acttgaTAGGGAAAGCTGAGGTATATG |  |
| CG3776 tag (set2F & set2R) | TGTAAAGGAGTTCACCGCTGGACAC | CCTGCTAGCTTACGTCACCACTTTG | 133 |
| CG3997 | CACAAGTCGTTCAGAATAAAGCAGAAGC | TGACGGCGCTTAGCGTTGTAACGA | 119 |
| CG4692 | GCCCTTCGGCCAGGTCAAGC | CTTTGGGAACACGTACTTGTGCTGC | 130 |
| CG5972 | AAATGAAACTGGCGGTCAATGCCAG | ACCGGGTCCATACTGGTTGCCT | 91 |
| CG7939 (RpL32) | CATCCGCCCAGCATACAG CCATTTGTGCGACAGCTTAG | CCATTTGTGCGACAGCTTAG | 97 |
| CG8108 | AGTTCACTCACCACAACTCGAGCA | CGTTTGCGATCATCGCTGCGGTC | 103 |
| CG9042 (GAPDH) | CGTCAAGTACCTGAAAGGACACAAGC | CGAAGATCAGGATGTCAGCGTTCTTG | 95 |
| CG13410 | AGAAGAGCACCTGCGTTTTGTATGGA | CAAACGCTTCGCAGCGCGCTT | 99 |
| dT.anchor for PAT: | GCGAGCTCCGCGGCCGCGTTTTTTTTTTTT | NA | NA |
| RpS2 PAT assay | CCTCGTCTGCACGCCGATGCCTAAGT | NA | 116+ |
| CG1349 PAT assay 1 | GGTCTTCTTGTGGCCTACAACTAACA | NA | 142+ |
| CG1349 PAT assay 2 | GCAAGGAGAAAGTCCAGGAGGT | NA | 171+ |
| CG3776 PAT assay | CATATAACATCGGCCCATGGCTA | NA | 154+ |
| CG3997 PAT assay | CTGTAAGCTGTTGATTCCAGGAG | NA | 127+ |

**Supplementary Table 8.** List of primers and oligos used in this study. The plus sign (+) for the sizes of the poly(A) length assay products indicates the smear, because the annealing of dT.anchor primer is random on the poly(A) tail.

**Supplementary Figures**


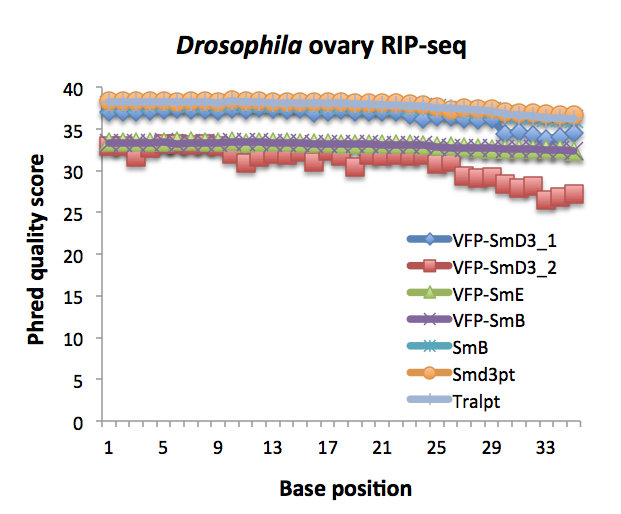

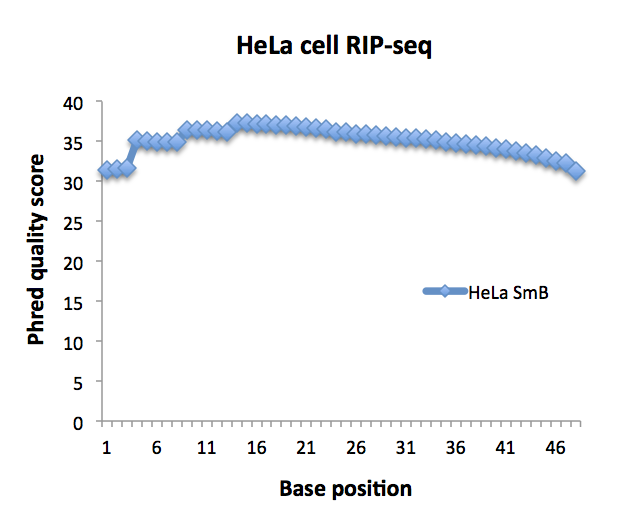


Supplementary Figure 1. Per base quality of the *Drosophila* (35nt) and human (48nt) RIP-seq data calculated using FastQC (reads are pooled for each experiment).


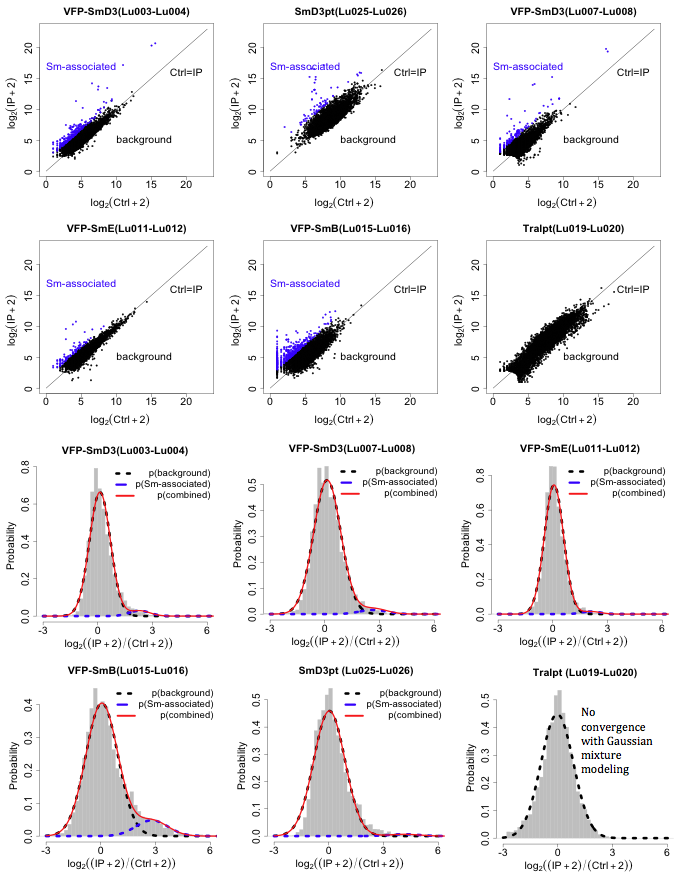


Supplementary Figure 2. Additional Scatterplots and Gaussian mixture modeling plots (related to Fig. 2a,b)


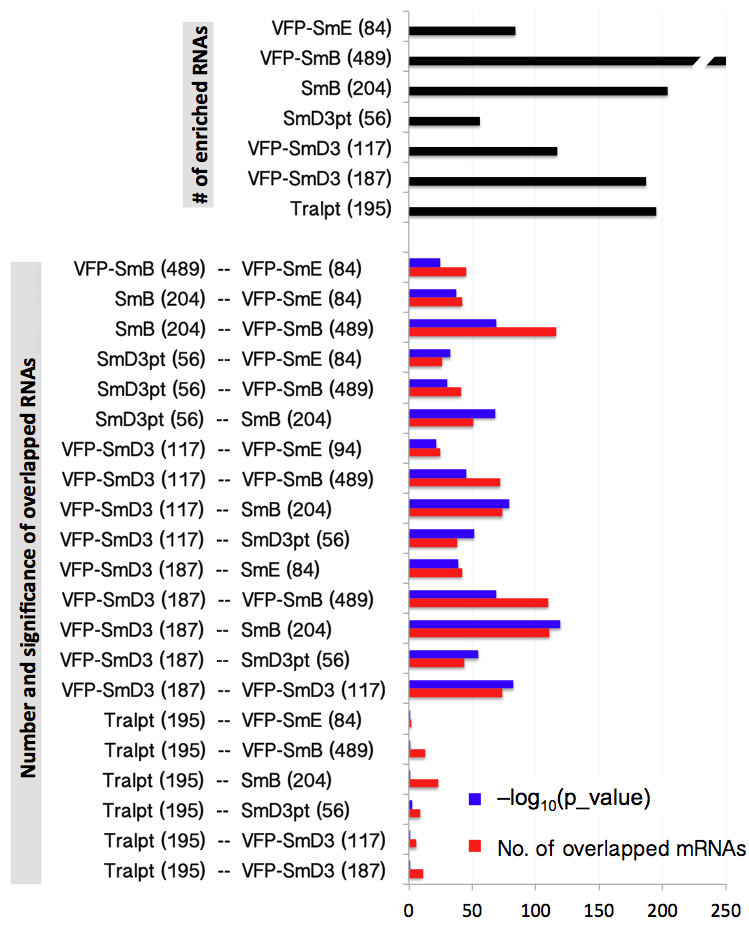


Supplementary Figure 3. Comparisons among all RIP-seq experiments, excluding ncRNAs (related to Fig. 2d).

Numbers of enriched RNAs are listed next to the experiment labels. Black bars: number of enriched RNAs in each experiments; red bars: number of overlapped RNAs in each pair; blue bars: negative log10 transformed Fisher’s exact test p-values (within a superset of 5270 RNAs).

**Supplementary Figure 4.** Enrichment ratios of the consensus set of Sm-associated RNAs, plotted by experiment and priming methods (related to Fig. 3). Tralpt is used as control.

U1_21D ATACTTACCTGGCGTAGAGGTTAACCGTGATCACGAAGGCGGTTCCTCCGGAGTGAGGCTTGGCCATTGCACCTCGGCTGAGTTGACCTCTGCGATTATT 100

U1_95Ca ATACTTACCTGGCGTAGAGGTTAACCGTGATCACGAAGGCGGTTCCTCCGGAGTGAGGCTTGGCCATTGCACCTCGGCTGAGTTGACCTCTGCGATTATT 100

U1_95Cb ATACTTACCTGGCGTAGAGGTTAACCGTGATCACGAAGGCGGTTCCTCCGGAGTGAGGCTTGGCCATTGCACCTCGGCTGAGTTGACCTCTGCGATTATT 100

U1_95Cc ATACTTACCTGGCGTAGAGGTTAACCGTGATCACGAAGGCGGTTCCTCCGGAGTGAGGCTTGGCCATTGCACCTCGGCTGAGTTGACCTCTGCGATTATT 100

U1_82Eb ATACTTACCTGGCGTAGAGGTTAACCGTGATCACGAAGGCGGTTCCTCCGGAGTGAGGCTTGGCCATTGTACCTCGGCTGAGTTGACCTCTGCGATTATT 100

********************************************************************* ******************************

U1_21D CCTAATGTGAATAACTCGTGCGTGTAATTTTTGGTAGCCGGGAATGGCGTTCGCGCCGTCCCGA 164

U1_95Ca CCTAATGTGAATAACTCGTGCGTGTAATTTTTGGTAGCCGGGAATGGCGTTCGCGCCGTCCCGA 164

U1_95Cb CCTAATGTGAATAACTCGTGCGTGTAATTTTTGGTAGCCGGGAATGGCGTTCGCGCCGTCCCGA 164

U1_95Cc CCTAATGTGAATAACTCGTGCGCGTAATTTTTGGTAGCCGGGAATGGCGTTCGCGCCGTCCCGA 164

U1_82Eb CCTAATGTGAATAACTCGTGCGTGTAATTTTTGTTAGCCGGGAATGGCGTTCGCGCCGTCCCGA 164

********************** ********** ******************************

U2_38Aba ATCGCTTCTCGGCCTTATGGCTAAGATCAAAGTGTAGTATCTGTTCTTATCAGCTTAACATCTGATAGTTCCTCCATTGGAGGACAACAAATGTTAAACT 100

U2_38ABb ATCGCTTCTCGGCCTTATGGCTAAGATCAAAGTGTAGTATCTGTTCTTATCAGCT-AACATCTGATAGTTCCTCCATTGGAGGACAACAAATGTTAAACT 99

U2_14B ATCGCTTCTCGGCCTTATGGCTAAGATCAAAGTGTAGTATCTGTTCTTATCAGCTTAACATCTGATAGTTCCTCCATTGGAGGACAACAAATGTTAAACT 100

U2_34ABc ATCGCTTCTCGGCCTTATGGCTAAGATCAAAGTGTAGTATCTGTTCTTATCAGCTTAACATCTGATAGTTCCTCCATTGGAGGACAACAAATGTTAAACT 100

U2_34ABb ATCGCTTCTCGGCCTTATGGCTAAGATCAAAGTGTAGTATCTGTTCTTATCAGCTTAACATCTGATAGTTCCTCCATTGGAGGACAACAAATGTTAAACT 100

U2_34ABa ATCGCTTCTCGGCCTTATGGCTAAGATCAAAGTGTAGTATCTGTTCTTATCAGCTTAACATCTGATAGTTCCTCCATTGGAGGACAACAAATGTTAAACT 100

******************************************************* ********************************************

U2_38ABa GATTTTTGGAATCAGACGGAGTGCTAGGGGCTTGCTCCACCTCTGTCACGGGTTGGCCCGGTATTGCAGTACCGCCGGGATTTCGGCCCAAC 192

U2_38ABb GATTTTTGGAATCAGACGGAGTGCTAGGGGCTTGCTCCACCTCTGTCACGGGTTGGCCCGGTATTGCAGTACCGCCGGGATTTCGGCCCAAC 191

U2_14B GATTTTTGGAATCAGACGGAGTGCTAGGGGCTTGCTCCACCTCTGTCACGGGTTGGCCCGGTATTGCAGTACCGCCGGGACTTCGGCCCAAC 192

U2_34ABc GATTTTTGGAATCAGACGGAGTGCTAGGAGCTTGCTCCACCTCTGTCGCGGGTTGGCCCGGTATTGCAGTACCGCCGGGATTTCGGCCCAAC 192

U2_34ABb GATTTTTGGAATCAGACGGAGTGCTAGGAGCTTGCTCCACCTCTGTCGCGGGTTGGCCCGGTATTGCAGTACCGCCGGGATTTCGGCCCAAC 192

U2_34ABa GATTTTTGGAATCAGACGGAGTGCTAGGGGCTTGCTCCACCTCTGTCGCGGGTTGGCCCGGTATTGCAGTACCGCCGGGATTTCGGCCCAAC 192

****************************.******************.******************************** ***********

U4_38AB ATCTTTGCGCAGAGGCGATATCGTAACCAATGAAG-TTCTACTGAGGTGCGATTATTGCTAGTTGAAAACTTTAACCAATACCCCGCCATGGGGACGTGA 99

U4_39B ATCTTTGCGCAGTGGCAATACCGTAACCAATGAAG-TCCTCCTGAGGTGCGGTTATTGCTAGTTGAAAACTTTAACCAATACCCCGCCATGGGGACGTGA 99

U4_25F AACCTTGTGCAGTGGCAACATCGCAAGCAATGAAGTTCCAACTGAGCTGCGATTATTGCTAGTTGAAAACTAAAACCAATATCTCGCCCAGCGTAAG-GA 99

*:* *** ****:***.* * ** ** ******** * *:.***** ****.*******************::******** * ****.:* * *.* **

U4_38AB AATACCGTC----CACTACGGCAATTTTTGGAAG-CCCGAGAGGGCCA- 142

U4_39B AATACCGTC----CACTACGGCAATTTTTGGAAG-CCCGAGAGGGCTAA 143

U4_25F TCTACGATCTTTAAGCTAAGGCAATTTTTTTAGGCCCCAAGTGGGCTGA 148

:.*** .** ..***.********** *.* ***.**:**** .

U5_23D ACTCTGGTTTCTCTTCAATTGTCGAATAAATCTTTCGCCTTTTACTAAAGATTTCCGTGGAGAGGAACACTCTAATGAGTCTAAACACAATTTTTGCTTA 100

U5_38ABb ACTCTGGTTTCTCTTCAATTGTCGAATAAATCTTTCGCCTTTTACTAAAGATTTCCGTGGAGAGGAACACTCTAATGAGTCTAAACACAATTTTT--ATT 98

U5_38ABa ACTCTGGTTTCTCTTCAATTGTCGAATAAATCTTTCGCCTTTTACTAAAGATTTCCGTGGAGAGGAACACTCTAATGAGTCTAAACTCAATTTTTG---T 97

U5_34A ACTCTGGTTTCTCTTCAATTGTCGAATAAATCTTTCGCCTTTTACTAAAGATTTCCGTGGAGAGGAACACTCTAATGAGTCTAAAATAATCTTTTG---T 97

U5_35D ACTCTGGTTTCTCTTCAATTGTCGAATAAATCTTTCGCCTTTTACTAAAGATTTCCGTGGAGAGGAACACTCTAATGAGTCTAAAATATTATTTTG---T 97

U5_14B ACTCTGGTTTCTCTTCAATTGTCGAATAAATCTTTCGCCTTTTACTAAAGATTTCCGTGGAGAGGAACACTCTAATGAGTCTAAACACAATTTTTT-ATT 99

U5_63BC ACTCTGGTTTCTCTTCAATTGTCGAATAAATCTTTCGCCTTTTACTAAAGATTTCCGTGGAGAGGAACACTCTAATGAGTCTAAA-ATAATTTTTA-GTA 98

************************************************************************************* : :: **** :

U5_34A AGTG-CCCGGCGACTTCGGTAGC-----TGGG-CCA- 129

U5_35D AGTG-CCCGGCGACTTTGGTAAC-----TGGG-CCA- 127

U5_63BC -GTG-CCCTGTCGC----AAGAC-----TGGGGCCA- 122

U5_38ABa -ATGACCTGGCTAAATATTTAGT-----TGGG-CCA- 126

U5_38ABb -GAGGCCTGATAACTT--ATG-CT---ATCGGGCCA- 126

U5_14B -GAGGCCTGATAACTT--ATG-TT---ATCGGGCCCA 129

U5_23D -GAGCCCCGATGGCAT--TTGCCT---TTGGGGCCA- 128

* ** * ** **

**Supplementary Figure 5.** Sequence alignment of *D. melanogaster* U1, U2, U4 and U5 paralogs. The paralogs of U1 and U2 have very few nucleotide variations and they are highlighted. U4 and U5 paralogs have significant differences among them.

Supplementary Figure 6 (next page). Characterization of scaRNA:Prp8. **a.** The scaRNA:prp8 is located in the conserved 4^th^ intron of Prp8 gene, and a previously identified microRNA mir-988 is located in the conserved 8^th^ intron. **b.** Sequence structure alignment of scaRNA:Prp8 in 13 selected insect species by LocARNA (global standard alignment). Blue box: H box; black box: ACA box; green boxes: CAB boxes; black half brackets: covariant basepairs. Species name abbreviation: *D.ere: D. erecta; D.pse: D. pseudoobscura; D.per: D.persimilis; D.wil: D. willistoni; D.vir: D. virilis; D.moj: D. mojavensis; D.gri: D. grimshawi; A.gam: Anopheles gambiae; A.mel: Apis mellifera*. **c.** Predicted secondary structure of the scaRNA:Prp8. **d.** Potential basepairing between the putative peudouridylation guide sequence and target snRNAs.

**a b**

**
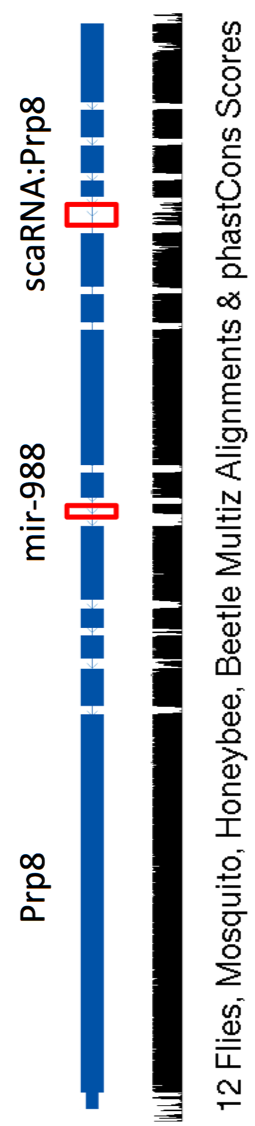
**


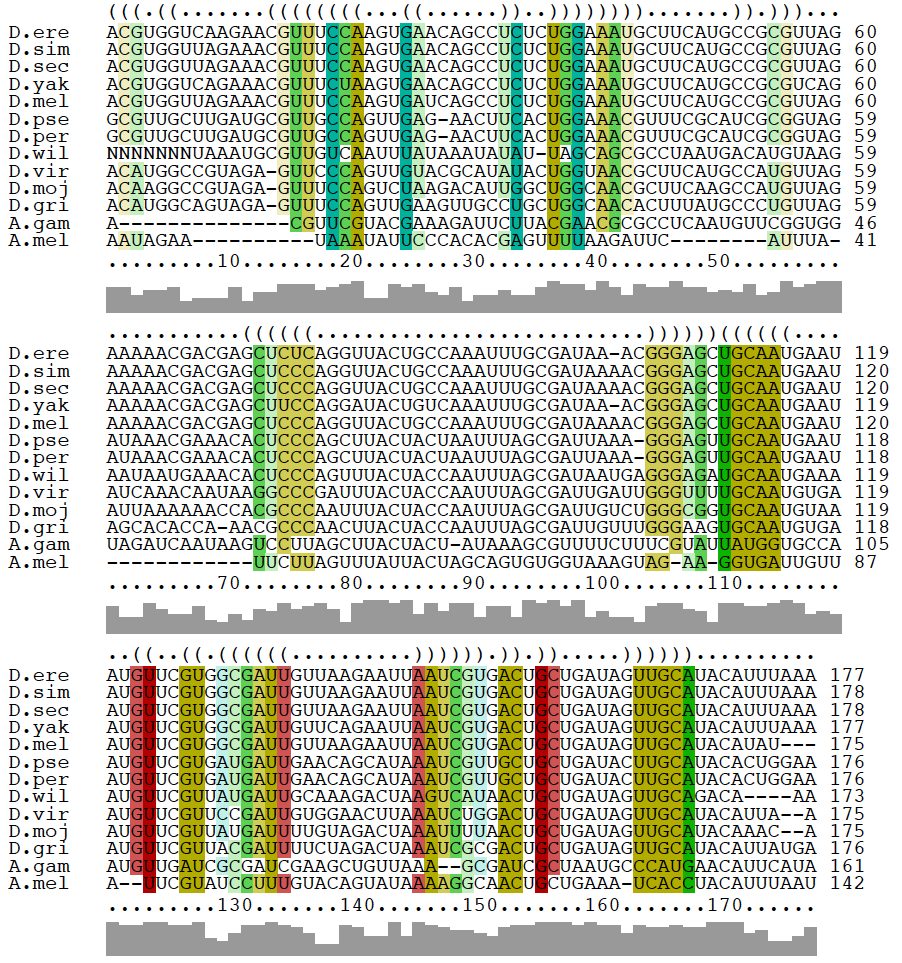

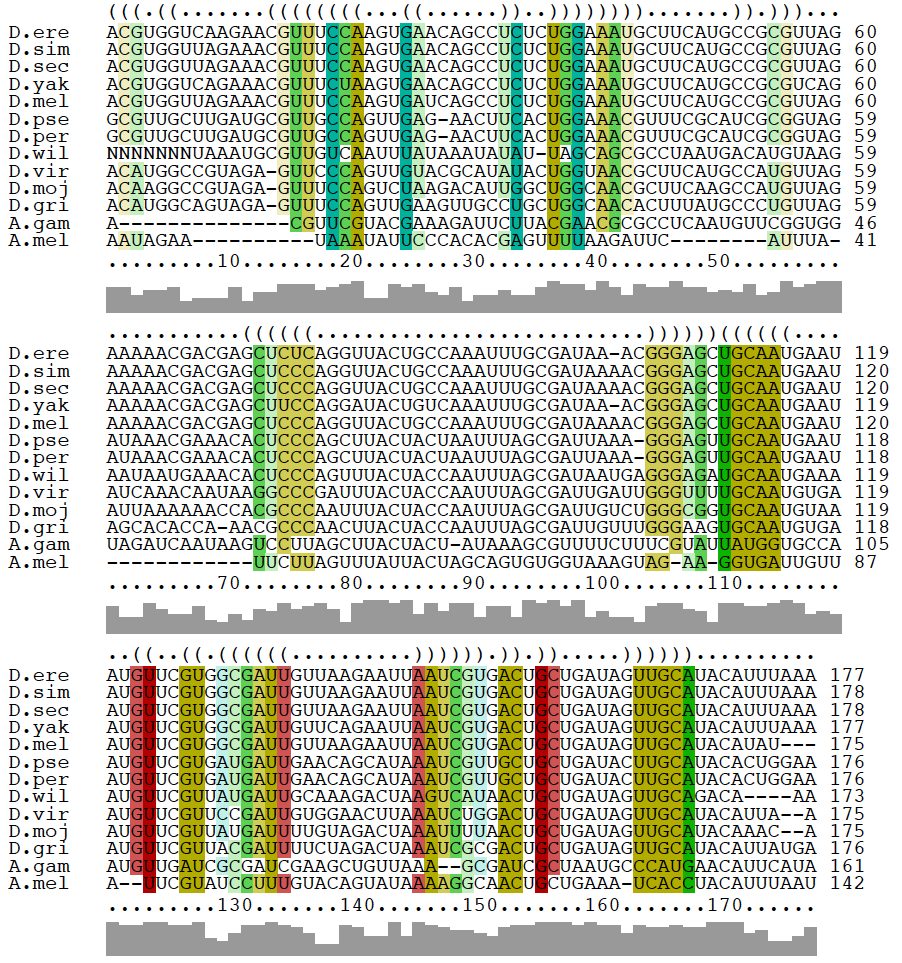

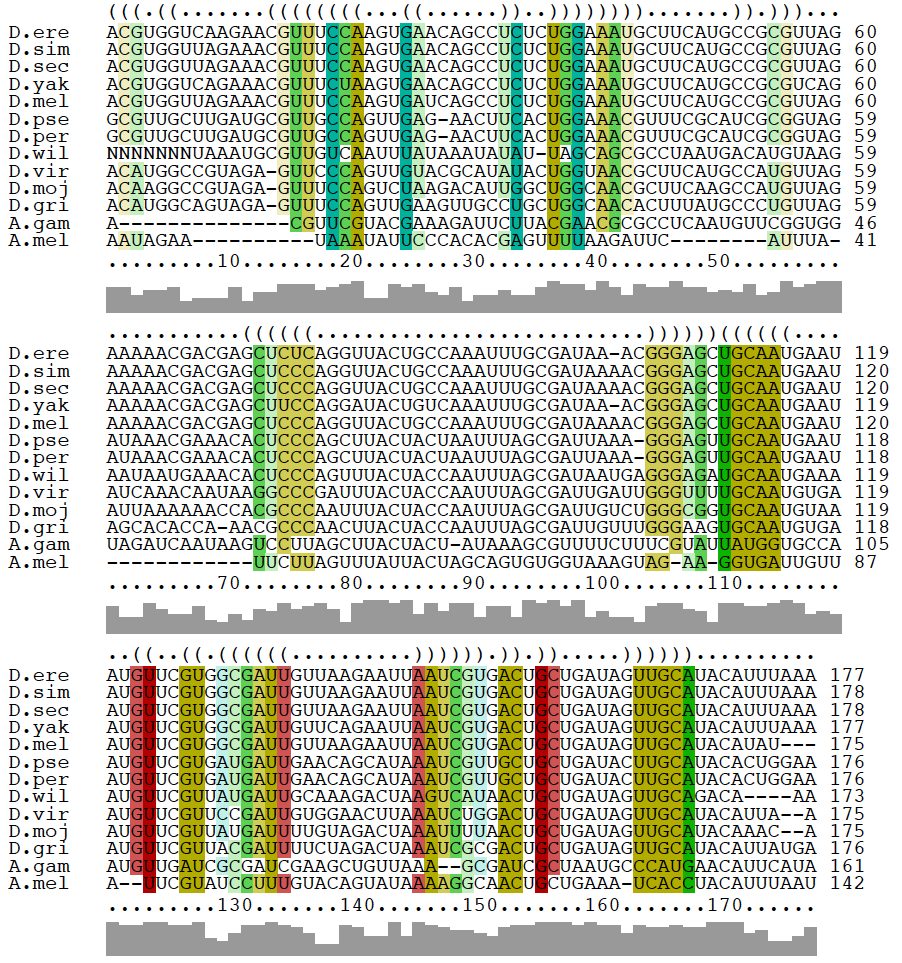


**c d**

**
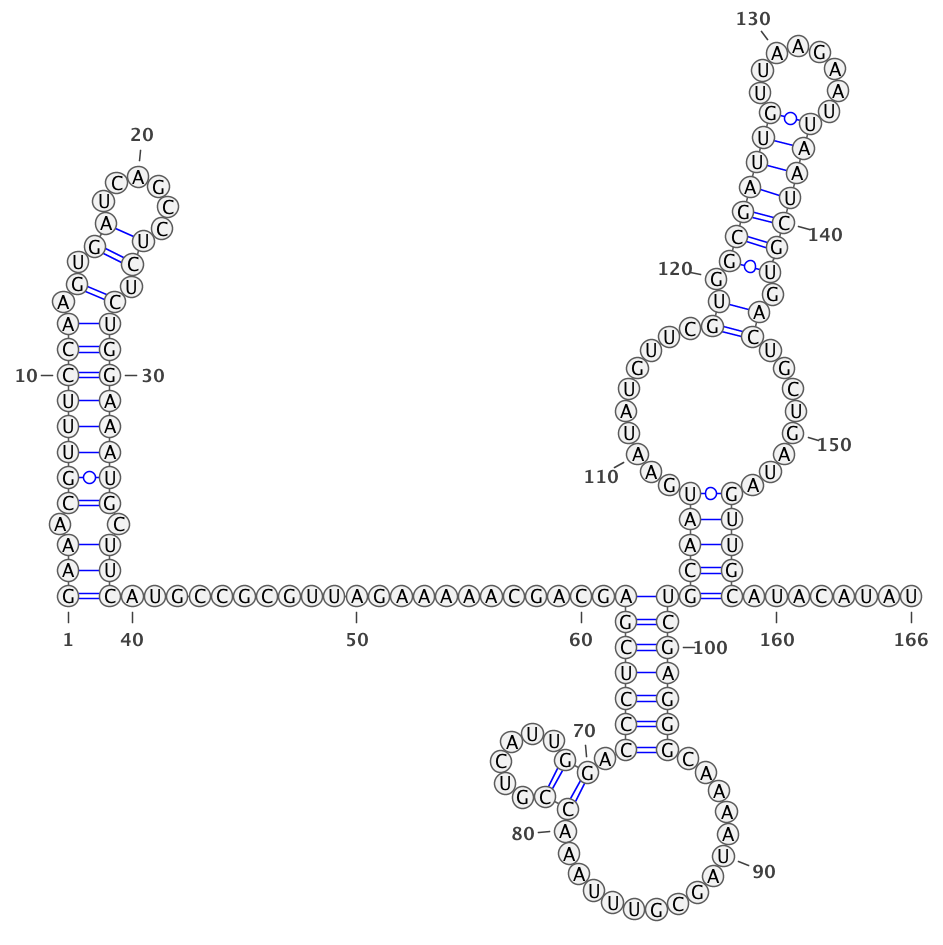

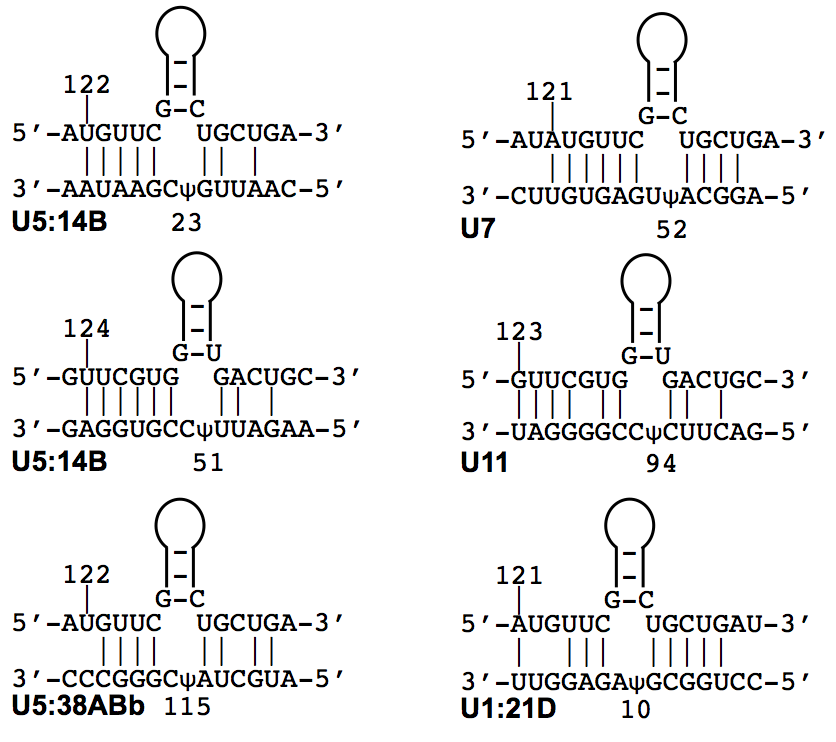
**

**Supplementary Figure 6.** Characterization of scaRNA:Prp8.

**
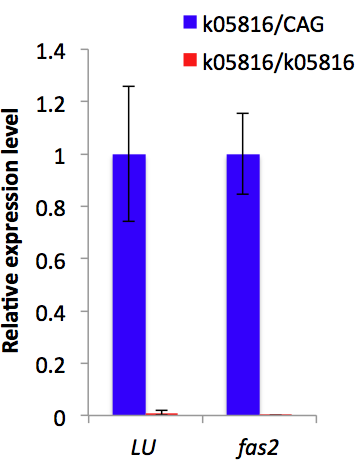
**

**PSEA PSEB**


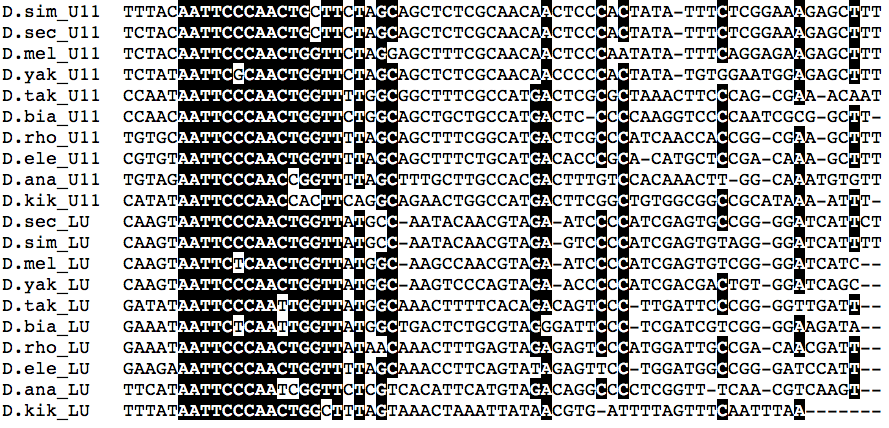


**a**

**b**


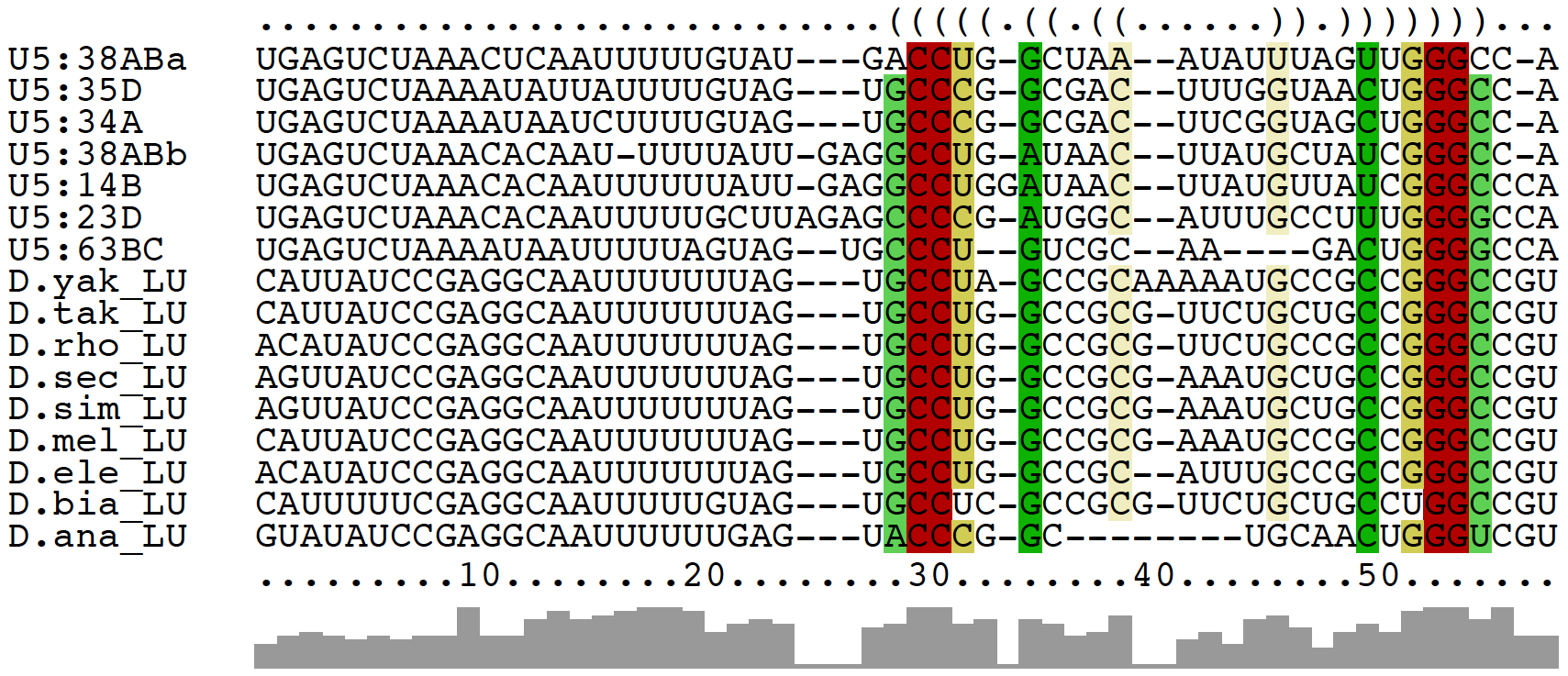


**d**

**e**


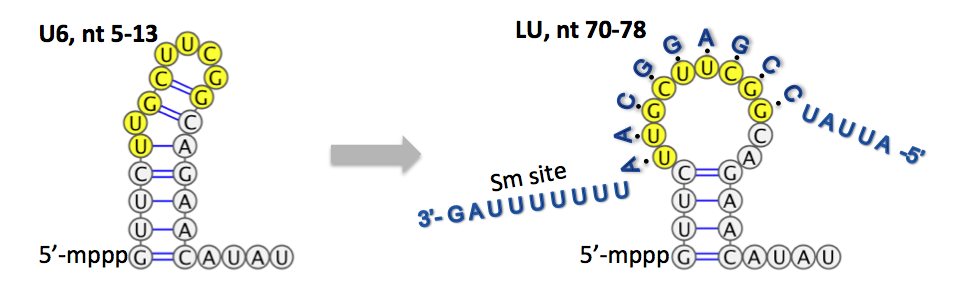

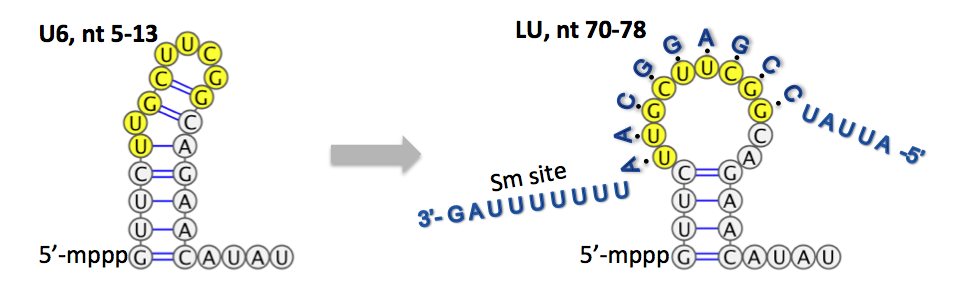


**U6**

**c**

D.mel_LU ATGTCTCGATCGCCGCTTCAGTTGTGGAGCGAGAGCTTACGCAATGGAGCGGAGTGATGA 60

D.mel_LU-p -----ACGATCGCCGCTTCAGTTGTGGAGCGATAGCTTACGCAATGGAGCGGAGTGATGA 55

************************** ***************************

D.mel_LU GCACATTATCCGAGGCAATTTTTTTAGTGCCTGGCCGCGAAATGCCGCCGGGCCGT 116

D.mel_LU-p GGATATTATCCGAGGCAAT------------------------------------- 74

* * ***************

**Supplementary Figure 7**. **a**: Alignment of U11 and LU snRNA promoters in 10 *Drosophila* species that have the LU gene. Alignment by ClustalW2, coloring by the Color Align Conservation app in Sequence Manipulation Suite. Highlighted nucleotides are >70% identical in all sequences. PSEA element is highly conserved, but not the PSEB element. Species name abbreviation: *D.mel: Drosophila melanogaster; D.yak: D. yakuba; D.sec: D. sechellia; D.sim: D. simulans; D.tak: D. takahashii; D.rho: D. rhopaloa; D.ele: D. elegans; D.bia: D. biarmipes; D.ana: D. ananassae; D.kik: D. kikkawai.* **b** Sequence structure alignment of the 3’ end of *D. melanogaster* U5 paralogs and LU snRNA orthologs using LocARNA (global standard alignment). Note the conservation of the 3’ end stem loop. Blue box: Sm site. **c.** Sequence alignment of the LU with its pseudogene paralog residing in an intron of *Ac3* (chr2L:21644292-21644365) in *D. melanogaster*. **d** Putative base pairing between LU and U6. Only the 5’ end of U6 and the interal single stranded region in LU are shown. **e** Expression of LU and *fas2* RNA is reduced to less than 1% in the P element insertion line 10580, as determined by quantitative RT-PCR. CAG: CyO actin::GFP balancer.

**a b**


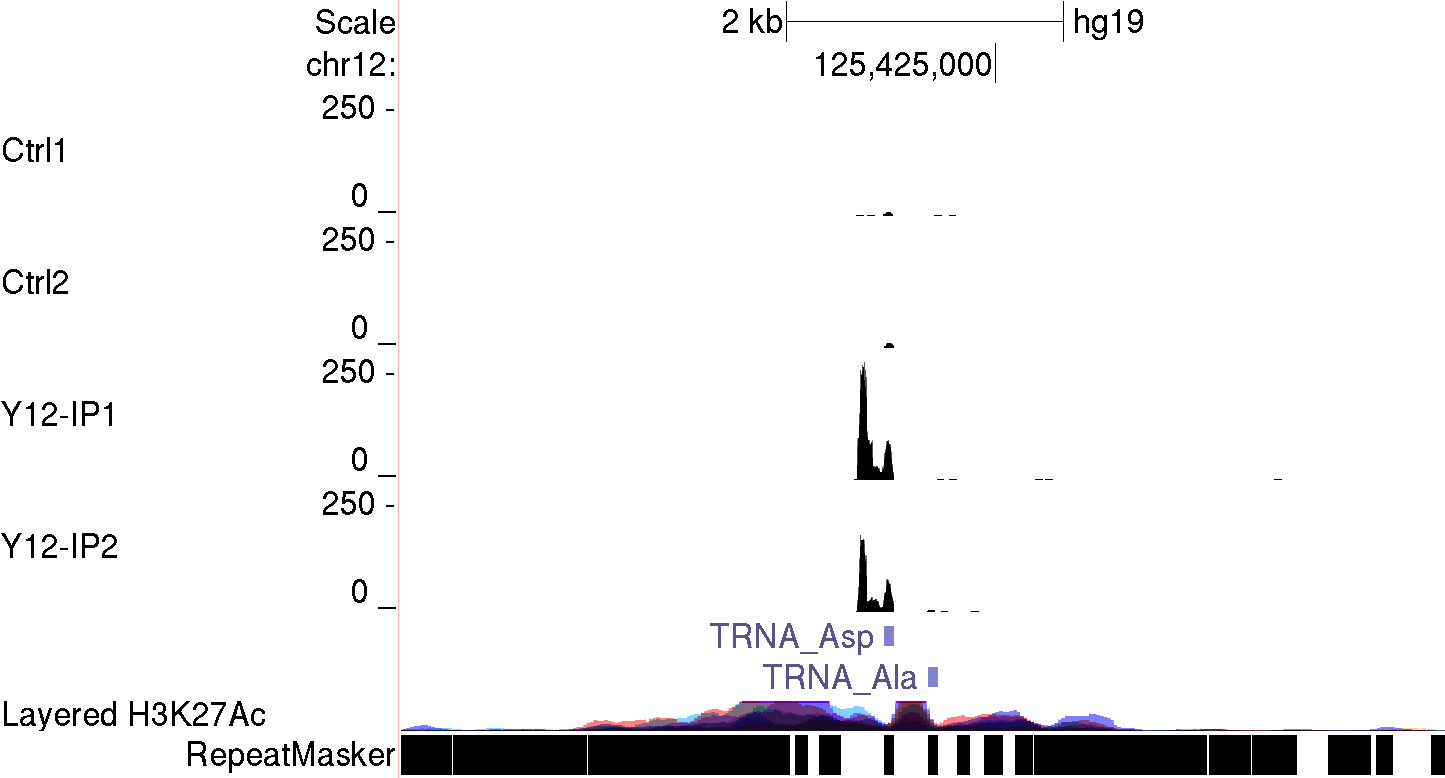

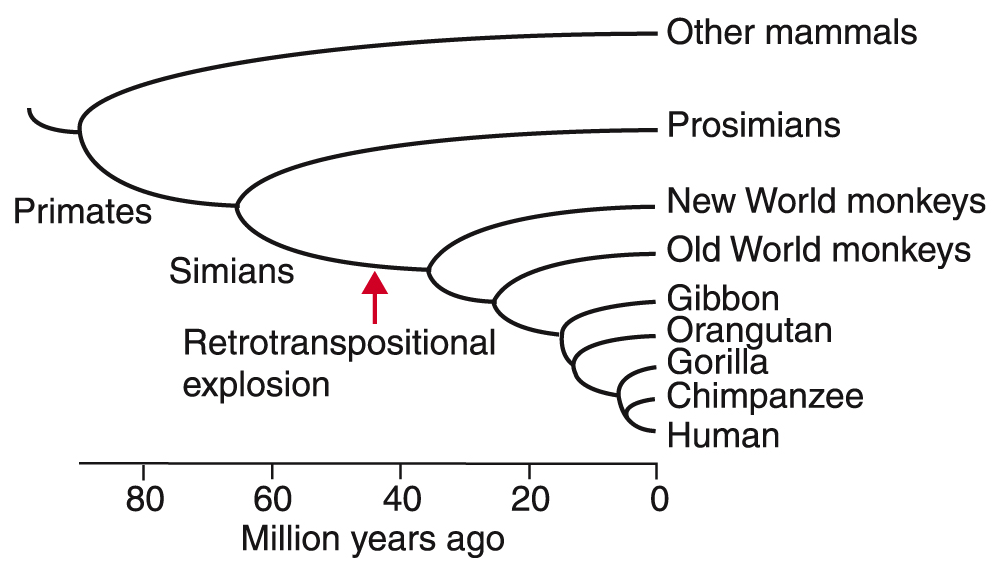


**c**
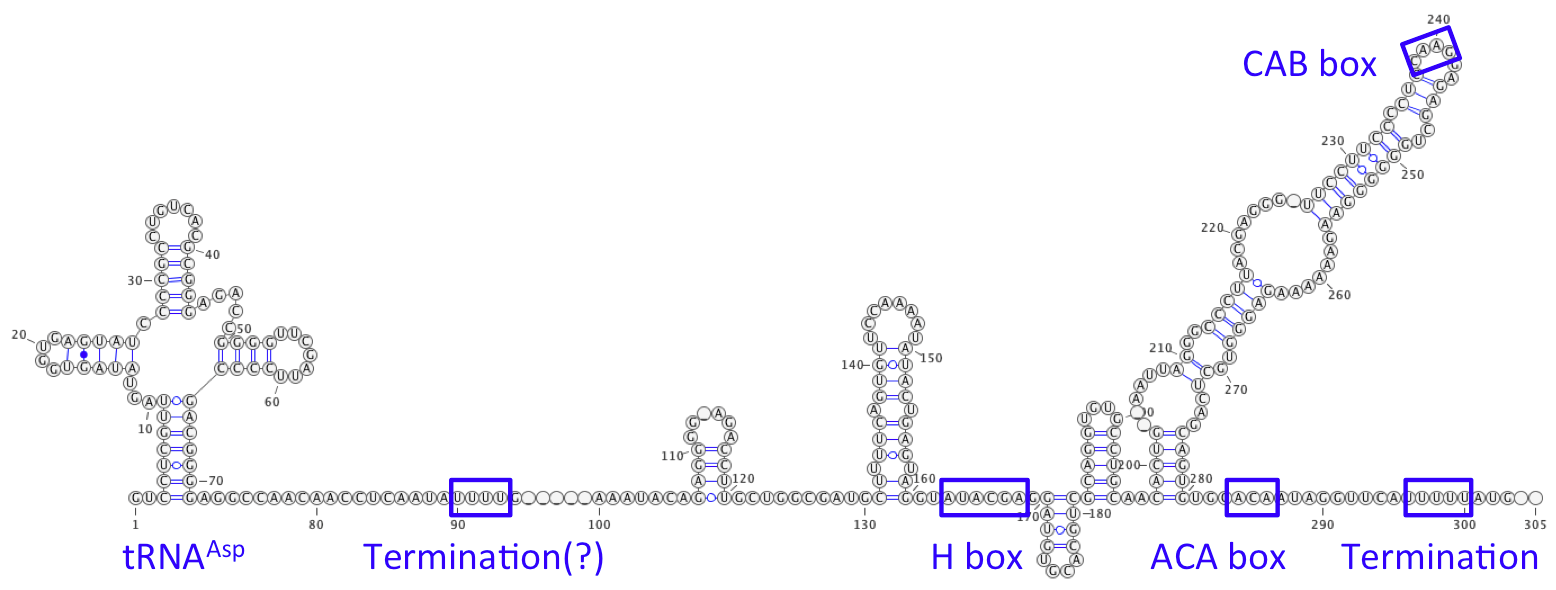
**_­_**

**Supplementary Figure 8.** Continued on the next page. Figure legend also on the next page.

**d**


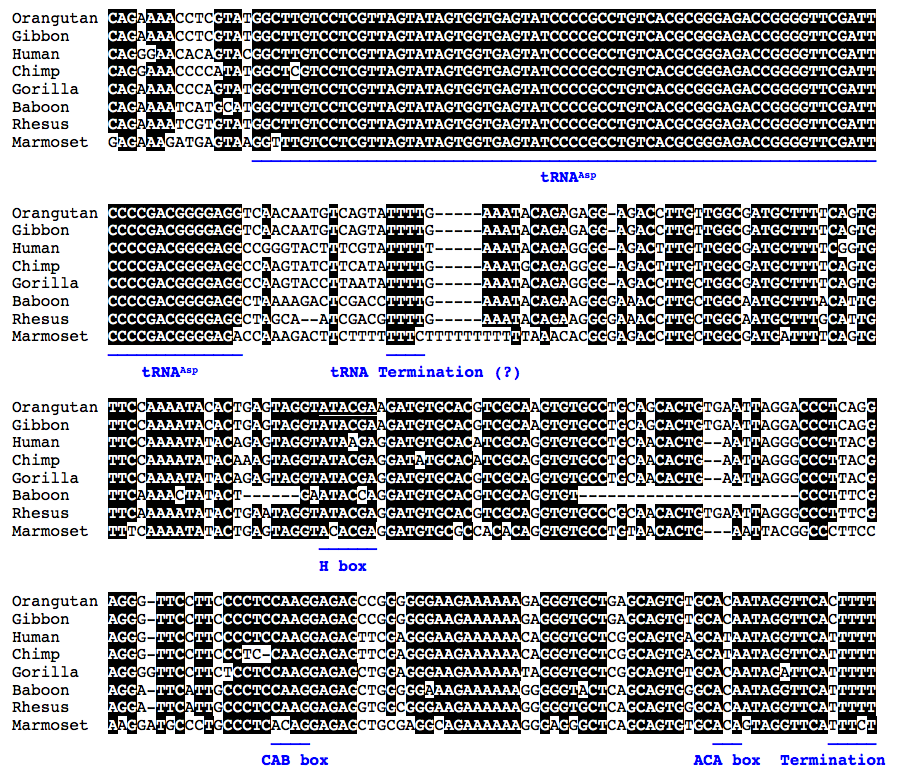


**Supplementary Figure 8.** Genome browser view, secondary structure, phylogeny and alignment of SHAN scaRNAs (related to Fig. 4b). **a**: Genome browser view of the SHAN locus, showing two Ctrls and two IPs. TRNA_Asp is part of the new gene SHAN, while TRNA_Ala is a separate tRNA gene. **b**: consensus secondary structure of SHAN orthologs. tRNA^Asp^: the tRNA part of this new gene SHAN; tRNA Termination (?): the presumed pol III transcription termination signal for tRNA; H/ACA/CAB boxes: putative scaRNAs sequence elements. Termination: putative pol III transcription termination signal for this new gene SHAN. **c**: SHAN scaRNA evolved from the root of simians (blue bracket), after the retrotranspositional explosion (reproduced from Ohshima et al., 2003 Genome Biology, published by Biomed Central). **d**: Alignment of SHAN scaRNAs orthologs from eight simian species. Human: *Homo* *sapiens*; Chimp: *Pan* *troglodytes*; Gorilla: *Gorilla* *gorilla*; Orangutan: *Pongo* *abelii*; Gibbon: *Nomascus* *leucogenys*; Rhesus: *Macaca* *mulatta*: macaque (Old World monkey); Baboon: *Papio anubis*  (Old World monkey); Marmoset: *Callithrix* *jacchus* (New World monkey).

_
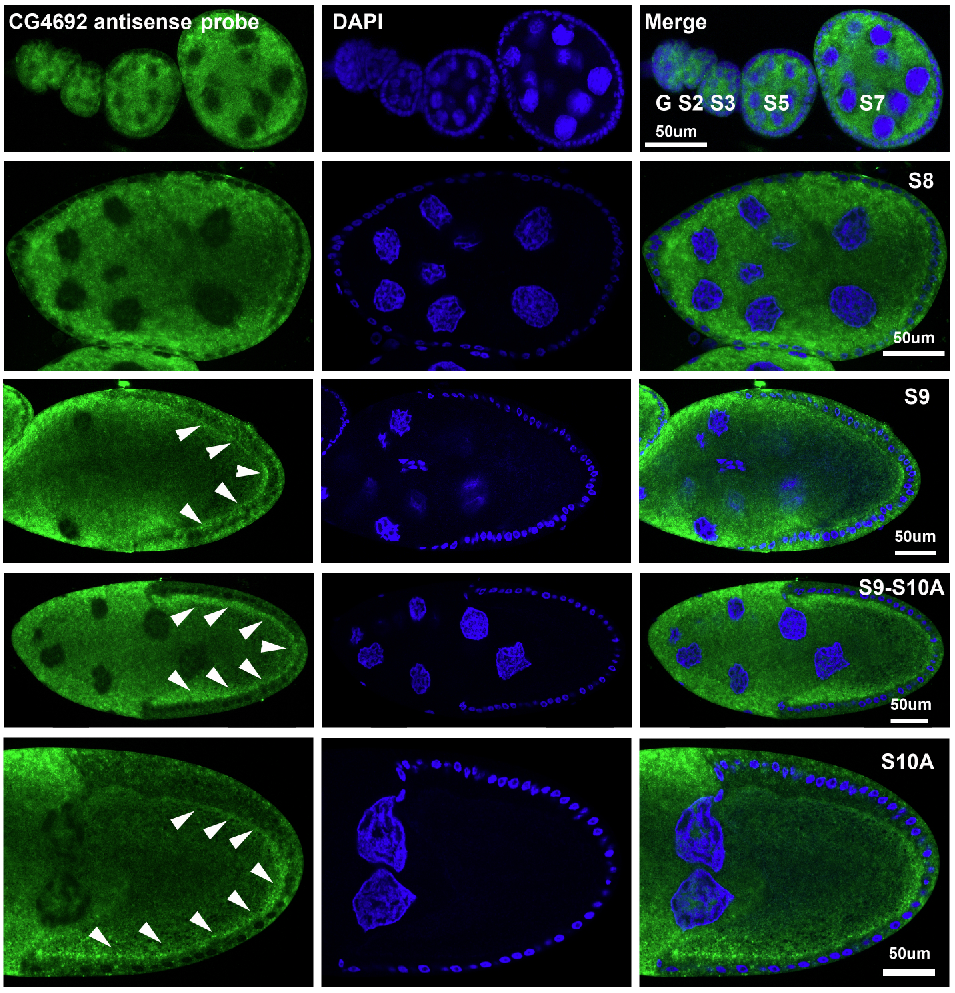

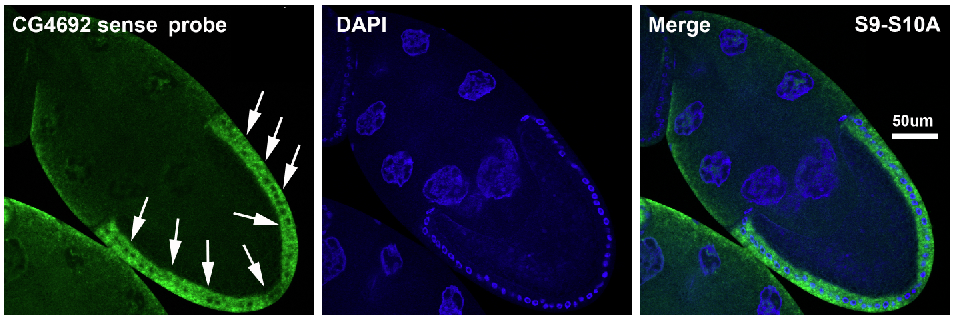
_

**Supplementary Figure 9.** CG4692 mRNA localizes along oocyte cortex. CG4692 mRNA (detected with CG4692 antisense probe) was enriched in the oocyte cortex from stages 9 to 10A (arrow heads, S9–S10A), but not in earlier stages (from germarium to stage 8). The CG4692 sense probe shows a localization pattern opposite to that of CG4692 mRNA (in follicle cells, arrows) and does not label the cortex. This latter pattern is likely due to the existence of a putative antisense transcript from the CG4692 locus (see EST data in Flybase.org).


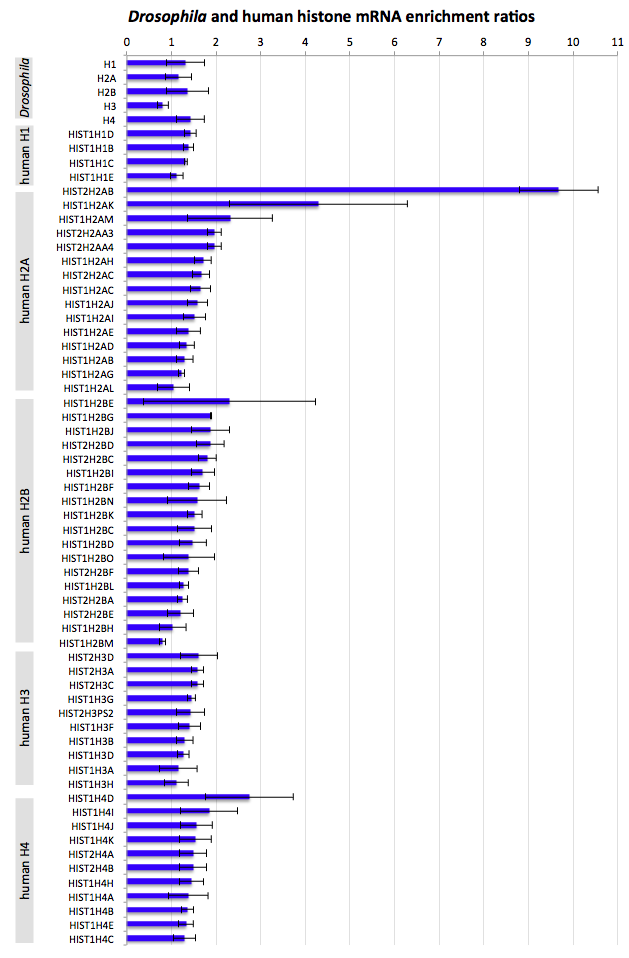


**Supplementary Figure 10.** Enrichment ratios for *Drosophila* and human replication-dependent histone mRNAs. Five *Drosophila* histone genes were displayed, summarizing all six *Drosophila* RIP-seq experiments. For the 86 human histone genes in the four clusters, 58 of them are detectable and shown in this figure. Error bars for *Drosophila* histone mRNAs represent standard deviation of enrichment ratios of six RIP-seq experiments, while human histone mRNA genes represent standard deviation of IP raw read numbers divided by the average of Ctrl raw read numbers. Note that most histone mRNAs are moderately, even though not significantly, enriched. Exact binomial test of all human histone mRNAs (58) gave a p-value of 4.1E-16, suggesting that human histone mRNAs are associated with Sm proteins (57 mRNAs with enrichment ratios > 1), even though most of the enrichment ratios are less than 2-fold and not significant.

**a b**


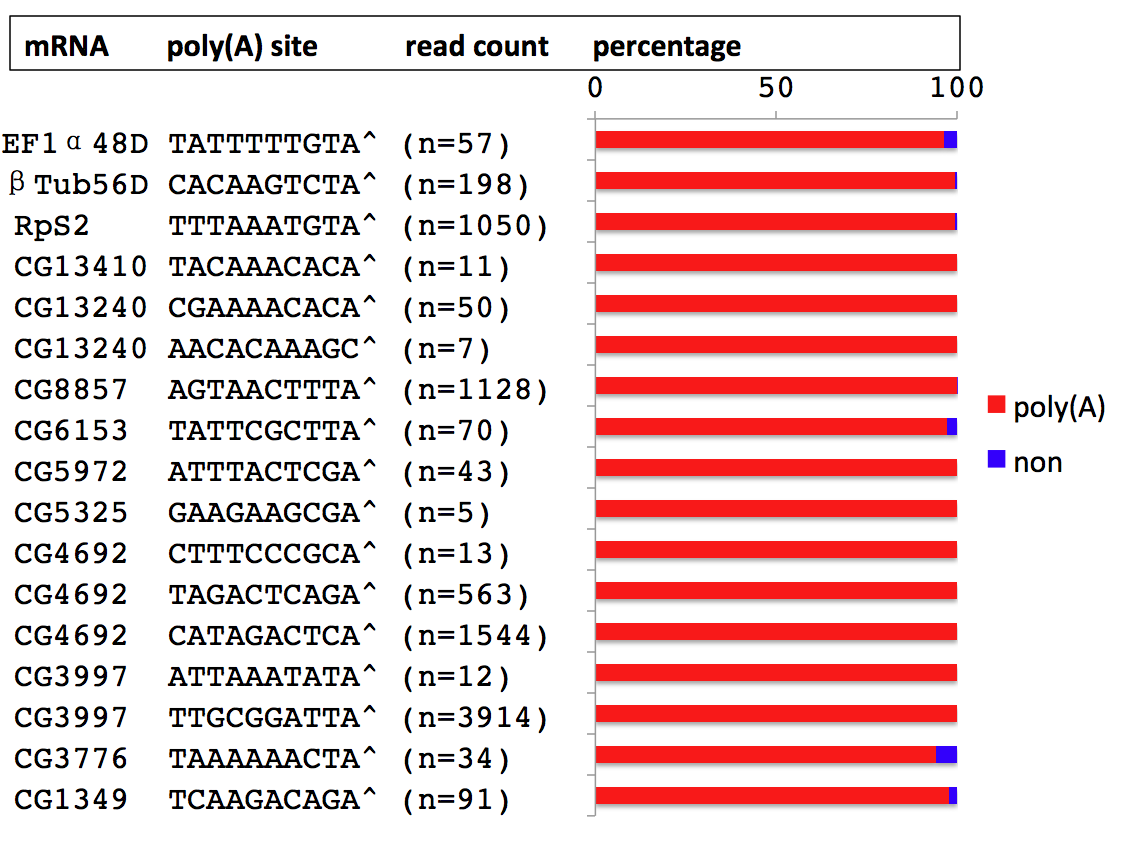

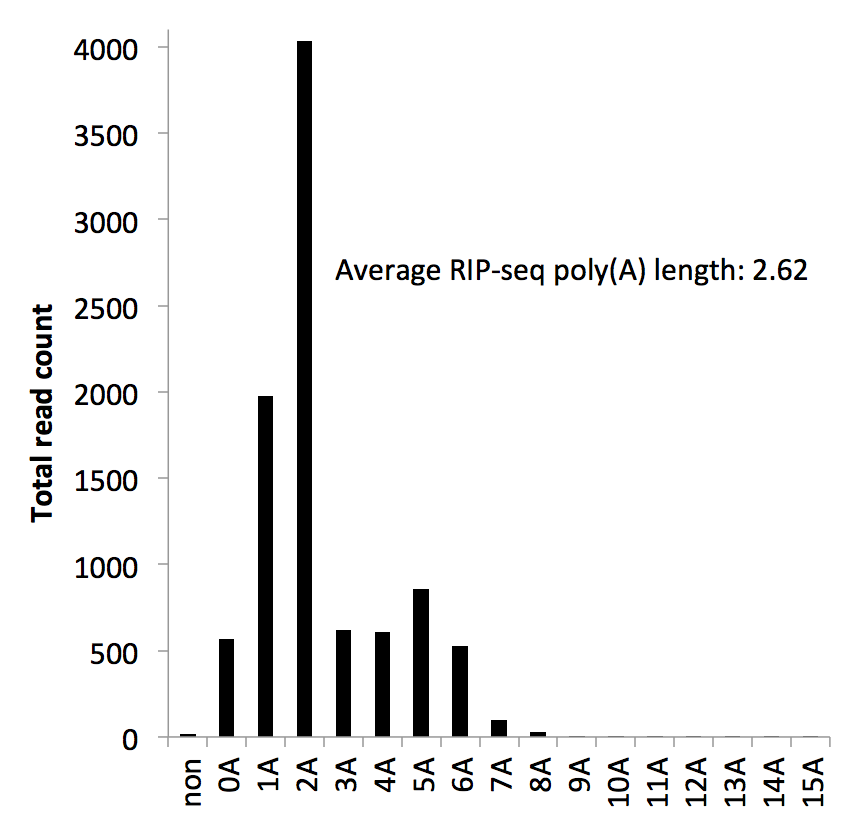


**Supplementary Figure 11.** Analysis of the polyadenylation of Sm-associated mRNAs. **a**. Percentage of polyadenylated and non-polyadenylated mRNAs that are associated with Sm proteins. Ten Sm-associated mRNAs were analyzed from the RIP-seq data. Some of them have multiple cleavage and polyadenylation sites. EF1α48D, βtub56D and RpS2 are used as control, non-Sm-associated mRNAs. **b**. Distribution of sequenced polyA lengths for the selected Sm-associated mRNAs in the RIP-seq data. Note: the lengths of sequenced polyA tails do not represent the real lengths of polyA tails, because the reads are short (35nt), and the ability of the sequencer to cover long homopolymer stretches is limited.


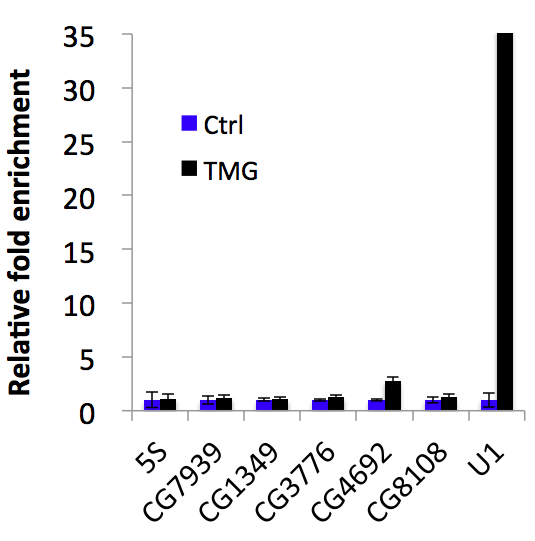


263 fold, std. = 67

p = 0.004

**Supplementary Figure 12.** Sm-associated mRNAs are not TMG-capped. Purified S2 cell total RNA was immunoprecipitated using TMG antibody (K121, unconjugated), and the immunoprecipitated RNA was measured using quantitative RT-PCR. Experiments were performed as quadruplicates and the error bars represent standard deviations. U1 snRNA was used as positive control. Std: standard deviation from four biological replicates.


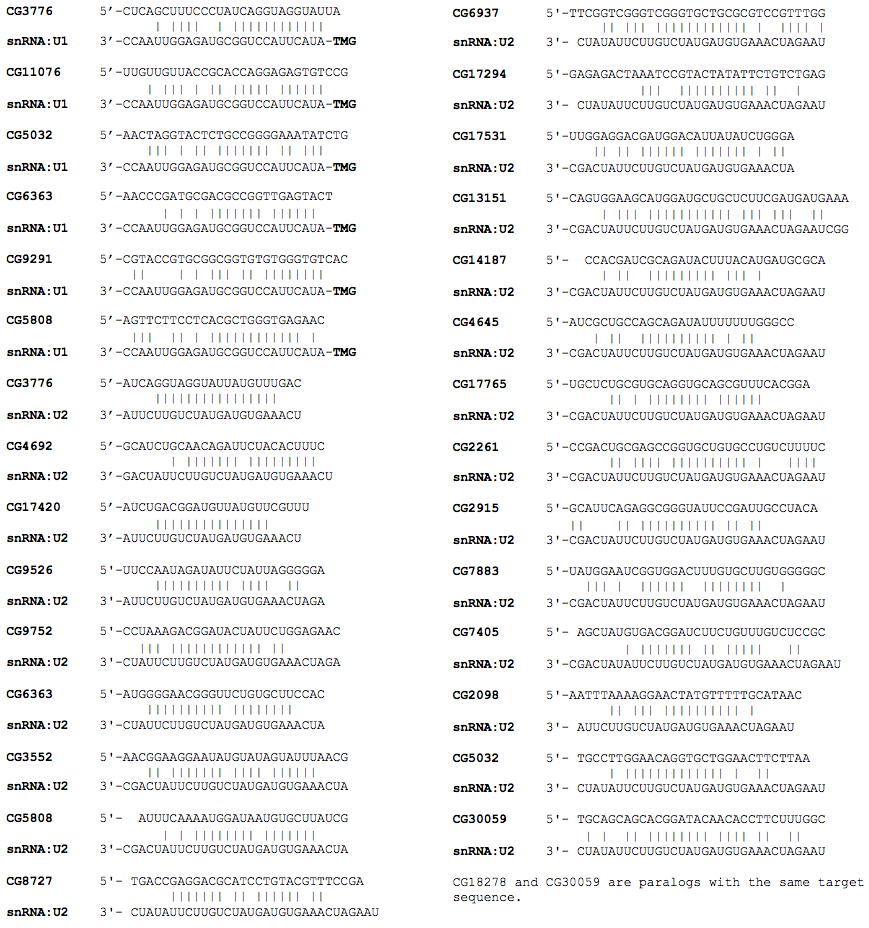


**Supplementary Figure 13**. Additional predicted snRNP-mRNA base pairings. These are only a subset of the most stable duplexes.
